# Supplementary material for: RAIN study: a protocol for a randomised controlled trial evaluating efficacy, safety and cost-effectiveness of intravenous-to-oral antibiotic switch therapy in neonates with a probable bacterial infection
Source: BMJ Open. 2019 Jul 9;9(7):e026688. doi: 10.1136/bmjopen-2018-026688 (PMC6615779; doi:10.1136/bmjopen-2018-026688)
Supplement: Supplementary data [file bmjopen-2018-026688supp001.pdf]

Supplementary material:

RAIN study: a protocol for a randomized controlled trial evaluating efficacy, safety and cost-effectiveness of intravenous-to-oral antibiotic switch therapy in neonates with a probable bacterial infection

1. SPIRIT checklist
2. Overview participating sites
3. Risk assessment tables for suspected early-onset bacterial infection from the Dutch national guideline: *NVK Richtlijn: Preventie en behandeling van early-onset neonatale infecties*
4. Questionnaires for assessment of cost-effectiveness (CEA) and Quality of Life (QoL).
5. Questionnaires for microbiome analysis

SPIRIT

STANDARD PROTOCOL ITEMS: RECOMMENDATIONS FOR INTERVENTIONAL TRIALS

SPIRIT 2013 Checklist: Recommended items to address in a clinical trial protocol and related documents*

Section/item	Item No	Description	Page
**Administrative information**			
Title	1	Descriptive title identifying the study design, population, interventions, and, if applicable, trial acronym	1
Trial registration	2a	Trial identifier and registry name. If not yet registered, name of intended registry	2 & 15
	2b	All items from the World Health Organization Trial Registration Data Set	yes
Protocol version	3	Date and version identifier	yes
Funding	4	Sources and types of financial, material, and other support	18
Roles and responsibilities	5a	Names, affiliations, and roles of protocol contributors	1
	5b	Name and contact information for the trial sponsor	1
	5c	Role of study sponsor and funders, if any, in study design; collection, management, analysis, and interpretation of data; writing of the report; and the decision to submit the report for publication, including whether they will have ultimate authority over any of these activities	N/A
	5d	Composition, roles, and responsibilities of the coordinating centre, steering committee, endpoint adjudication committee, data management team, and other individuals or groups overseeing the trial, if applicable (see Item 21a for data monitoring committee)	13 & 14
**Introduction**			

Background and rationale	6a	Description of research question and justification for undertaking the trial, including summary of relevant studies (published and unpublished) examining benefits and harms for each intervention	4-6
	6b	Explanation for choice of comparators	4-6
Objectives	7	Specific objectives or hypotheses	6
Trial design	8	Description of trial design including type of trial (eg, parallel group, crossover, factorial, single group), allocation ratio, and framework (eg, superiority, equivalence, noninferiority, exploratory)	7

Methods: Participants, interventions, and outcomes

Study setting	9	Description of study settings (eg, community clinic, academic hospital) and list of countries where data will be collected. Reference to where list of study sites can be obtained	7
Eligibility criteria	10	Inclusion and exclusion criteria for participants. If applicable, eligibility criteria for study centres and individuals who will perform the interventions (eg, surgeons, psychotherapists)	7
Interventions	11a	Interventions for each group with sufficient detail to allow replication, including how and when they will be administered	8
	11b	Criteria for discontinuing or modifying allocated interventions for a given trial participant (eg, drug dose change in response to harms, participant request, or improving/worsening disease)	8
	11c	Strategies to improve adherence to intervention protocols, and any procedures for monitoring adherence (eg, drug tablet return, laboratory tests)	10
	11d	Relevant concomitant care and interventions that are permitted or prohibited during the trial	N/A

Outcomes	12	Primary, secondary, and other outcomes, including the specific measurement variable (eg, systolic blood pressure), analysis metric (eg, change from baseline, final value, time to event), method of aggregation (eg, median, proportion), and time point for each outcome. Explanation of the clinical relevance of chosen efficacy and harm outcomes is strongly recommended	11
Participant timeline	13	Time schedule of enrolment, interventions (including any run-ins and washouts), assessments, and visits for participants. A schematic diagram is highly recommended (see Figure)	Figure is added
Sample size	14	Estimated number of participants needed to achieve study objectives and how it was determined, including clinical and statistical assumptions supporting any sample size calculations	10
Recruitment	15	Strategies for achieving adequate participant enrolment to reach target sample size	N/A

Methods: Assignment of interventions (for controlled trials)

Allocation:			
Sequence generation	16a	Method of generating the allocation sequence (eg, computer-generated random numbers), and list of any factors for stratification. To reduce predictability of a random sequence, details of any planned restriction (eg, blocking) should be provided in a separate document that is unavailable to those who enrol participants or assign interventions	8
Allocation concealment mechanism	16b	Mechanism of implementing the allocation sequence (eg, central telephone; sequentially numbered, opaque, sealed envelopes), describing any steps to conceal the sequence until interventions are assigned	8

Implementation	16c	Who will generate the allocation sequence, who will enrol participants, and who will assign participants to interventions	8
Blinding (masking)	17a	Who will be blinded after assignment to interventions (eg, trial participants, care providers, outcome assessors, data analysts), and how	N/A, page 8
	17b	If blinded, circumstances under which unblinding is permissible, and procedure for revealing a participant's allocated intervention during the trial	N/A

Methods: Data collection, management, and analysis

Data collection methods	18a	Plans for assessment and collection of outcome, baseline, and other trial data, including any related processes to promote data quality (eg, duplicate measurements, training of assessors) and a description of study instruments (eg, questionnaires, laboratory tests) along with their reliability and validity, if known. Reference to where data collection forms can be found, if not in the protocol	11
	18b	Plans to promote participant retention and complete follow-up, including list of any outcome data to be collected for participants who discontinue or deviate from intervention protocols	N/A
Data management	19	Plans for data entry, coding, security, and storage, including any related processes to promote data quality (eg, double data entry; range checks for data values). Reference to where details of data management procedures can be found, if not in the protocol	11
Statistical methods	20a	Statistical methods for analysing primary and secondary outcomes. Reference to where other details of the statistical analysis plan can be found, if not in the protocol	12
	20b	Methods for any additional analyses (eg, subgroup and adjusted analyses)	12
	20c	Definition of analysis population relating to protocol non-adherence (eg, as randomised analysis), and any statistical methods to handle missing data (eg, multiple imputation)	11

Methods: Monitoring

Data monitoring	21a	Composition of data monitoring committee (DMC); summary of its role and reporting structure; statement of whether it is independent from the sponsor and competing interests; and reference to where further details about its charter can be found, if not in the protocol. Alternatively, an explanation of why a DMC is not needed	12 & 16
	21b	Description of any interim analyses and stopping guidelines, including who will have access to these interim results and make the final decision to terminate the trial	11-12
Harms	22	Plans for collecting, assessing, reporting, and managing solicited and spontaneously reported adverse events and other unintended effects of trial interventions or trial conduct	16
Auditing	23	Frequency and procedures for auditing trial conduct, if any, and whether the process will be independent from investigators and the sponsor	N/A

Ethics and dissemination

Research ethics approval	24	Plans for seeking research ethics committee/institutional review board (REC/IRB) approval	17
Protocol amendments	25	Plans for communicating important protocol modifications (eg, changes to eligibility criteria, outcomes, analyses) to relevant parties (eg, investigators, REC/IRBs, trial participants, trial registries, journals, regulators)	N/A
Consent or assent	26a	Who will obtain informed consent or assent from potential trial participants or authorised surrogates, and how (see Item 32)	7-8
	26b	Additional consent provisions for collection and use of participant data and biological specimens in ancillary studies, if applicable	Mentioned in patient information form

Confidentiality	27	How personal information about potential and enrolled participants will be collected, shared, and maintained in order to protect confidentiality before, during, and after the trial	Mentioned in patient information form, page 14
Declaration of interests	28	Financial and other competing interests for principal investigators for the overall trial and each study site	20
Access to data	29	Statement of who will have access to the final trial dataset, and disclosure of contractual agreements that limit such access for investigators	A trial agreement has been made. Not mentioned in the manuscript.
Ancillary and post-trial care	30	Provisions, if any, for ancillary and post-trial care, and for compensation to those who suffer harm from trial participation	Mentioned in patient information form
Dissemination policy	31a	Plans for investigators and sponsor to communicate trial results to participants, healthcare professionals, the public, and other relevant groups (eg, via publication, reporting in results databases, or other data sharing arrangements), including any publication restrictions	16-17
	31b	Authorship eligibility guidelines and any intended use of professional writers	N/A
	31c	Plans, if any, for granting public access to the full protocol, participant-level dataset, and statistical code	N/A
**Appendices**			

Informed consent materials	32	Model consent form and other related documentation given to participants and authorised surrogates	Not attached, in dutch.
Biological specimens	33	Plans for collection, laboratory evaluation, and storage of biological specimens for genetic or molecular analysis in the current trial and for future use in ancillary studies, if applicable	12-15

2. Table 1: participating sites RAIN study

Amphia Hospital	Breda, The Netherlands
Erasmus MC-Sophia Children's Hospital	Rotterdam, The Netherlands
Groene Hart Hospital	Gouda, The Netherlands
Haaglanden Medical Center	Den Haag, The Netherlands
Haga/Juliana Children's Hospital	Den Haag, The Netherlands
Ikazia Hospital	Rotterdam, The Netherlands
Isala Klinieken	Zwolle, The Netherlands
Maasstad Hospital	Rotterdam, The Netherlands
Maxima Medical Center	Veldhoven, The Netherlands
Meander Medical Center	Amersfoort, The Netherlands
Medisch Spectrum	Twente, The Netherlands
Reinier de Graaf Hospital	Delft, The Netherlands
Rijnstate Hospital	Arnhem, The Netherlands
Sint Antonius Hospital *(2 locations)*	Nieuwegein & Utrecht, The Netherlands
Franciscus Gasthuis & Vlietland *(2 locations)*	Rotterdam & Schiedam, The Netherlands
IJsselland Hospital	Capelle aan den IJssel, The Netherlands

3. Risk assessment tables for suspected early-onset bacterial infection from the Dutch national guideline: *NVK Richtlijn: Preventie en behandeling van early-onset neonatale infecties*

Table 1: Risk factors for early-onset neonatal infection, including 'red flags

Risk factor	Red flag
**Parenteral antibiotic treatment given to the woman for confirmed or suspected invasive bacterial infection (such as septicaemia) at any time during labour, or in the 24-hour periods before and after the birth [This does not refer to intrapartum antibiotic prophylaxis]**	**yes**
**Suspected or confirmed infection in another baby in the case of a multiple pregnancy**	**yes**
Invasive group B streptococcal infection in a previous baby	
Maternal group B streptococcal colonisation, bacteriuria or infection in the current pregnancy	
Prelabour rupture of membranes >24 hours in term baby	
Preterm birth following spontaneous labour (before 37 weeks' gestation)	
Suspected or confirmed rupture of membranes for more than 18 hours in a preterm birth	
Intrapartum fever higher than 38°C, or confirmed or suspected chorioamnionitis	

Table 2 Clinical indicators of possible early-onset neonatal infection (observations and events in the baby), including 'red flags' Dutch national protocol. Adopted from the NICE guidelines 2012.

Clinical indicator	Red flag
**Respiratory distress starting more than 4 hours after birth**	**yes**
**Neonatal seizures**	**yes**
**Need for mechanical ventilation in a term baby**	**yes**
**Signs of shock**	**yes**
Altered behaviour or responsiveness (including floppiness)	
Feeding difficulties (including vomiting, excessive gastric aspirates and abdominal distension)	
Apnoea or bradycardia	
Signs of respiratory distress	
Hypoxia (for example, central cyanosis or reduced oxygen saturation level	
Signs of neonatal encephalopathy	
Need for cardio–pulmonary resuscitation	
Need for mechanical ventilation in a preterm baby	
Persistent fetal circulation (persistent pulmonary hypertension)	
Temperature abnormality (lower than 36°C or higher than 38°C) unexplained by environmental factors	
Signs of local infection (eyes, skin)	

References:

1. Neonatal infection (early onset): antibiotics for prevention and treatment [Internet]. 2012 [cited 28-08-2018]. Available from: https://www.nice.org.uk/guidance/cg149.

2. NVK Richtlijn: Preventie en behandeling van early-onset neonatale infecties, 2017. [cited 23-01-2019] Available from: https://www.nvk.nl/Kwaliteit/Richtlijnen-overzicht/Details/articleType/ArticleView/articleId/694/Preventie-en-behandeling-van-early-onset-neonatale-infecties#tab0

Survey 'ENGLISH: Survey day 7'

ENGLISH: Survey day 7 - Introduction

Dear parents/guardians, We have asked you permission to send two surveys as follow up on the RAIN study. This is the first survey, which has to be filled in around day 7 after initiation of therapy. Thank you for your help! RAIN team

ENGLISH: Survey day 7 - Survey day 7

Number	Question	Answers
1.1	Age mother	[ ] years
1.2	Age father	[ ] years
1.3	What is your highest degree in education?	○ I never finished school or training programme<br>○ Primary/Elementary school<br>○ Junior vocational eductaion<br>○ Lower genearal secondary education<br>○ Intermediate vocational education<br>○ Higher general secundary education<br>○ School for higher vocational education<br>○ University<br>○ Other degree
1.3.1	***If 'What is your highest degree in education?' is equal to 'Other degree' answer this question:***<br>If other, specify degree	[ ]
1.4	What is your highest degree in education?	○ I never finished school or training programme<br>○ Primary/Elementary school<br>○ Junior vocational eductaion<br>○ Lower genearal secondary education<br>○ Intermediate vocational education<br>○ Higher general secundary education<br>○ School for higher vocational education<br>○ University<br>○ Other degree
1.3.2	***If 'What is your highest degree in education?' is equal to 'Other degree' answer this question:***<br>If other, specify degree	[ ]

1.5	How many children (<18 years) do you have?	[ ] Children
1.6	Do they all live at home with you?	○ Yes<br>○ No
1.7	Do you, mother, have a paid job?	○ No<br>○ Yes, fulltime (>36 hours/week)<br>○ Yes, parttime
1.7.1	***If 'Do you, mother, have a paid job?' is equal to 'Yes, parttime' answer this question:***<br>If parttime, how many hours per week?	[ ] hours
1.7.2	***If 'Do you, mother, have a paid job?' is not equal to 'No' answer this question:***<br>Mother: What is (on average) your monthly salary (gross amount)?	○ Less than €999/month<br>○ Between €1.000 and 1.499/month<br>○ Between €1.500 and 1.999/month<br>○ Between €2.000 and 2.499/month<br>○ Between €2.500 and 2.999/month<br>○ Between €3.000 and 3.499/month<br>○ Between €3.500 and 3.999/month<br>○ Between €4.000 and 4.999/month<br>○ Between €5.000 and 5.999/month<br>○ Between €6.000 and 6.999/month<br>○ Between €7.000 and 7.999/month<br>○ Between €8.000 and 8.999/month<br>○ Between €9.000 and 9.999/month<br>○ More than €10.000/month<br>○ I don't know, I do not want to tell this
1.8	Do you, mother, have an unpaid job (except for caregiving)?	○ Yes<br>○ No
1.8.1	***If 'Do you, mother, have an unpaid job (except for caregiving)?' is equal to 'Yes' answer this question:***<br>if yes, for how many hours per month?	[ ]
1.9	Do you, father, have a paid job?	○ No<br>○ Yes, fulltime (>36 hours/week)<br>○ Yes, parttime
1.9.1	***If 'Do you, father, have a paid job?' is equal to 'Yes, parttime' answer this question:***<br>If parttime, how many hours per week?	[ ] hours

1.9.2	***If 'Do you, father, have a paid job?' is not equal to 'No' answer this question:***<br>Father: What is (on average) your monthly salary (gross amount)?	○ Less than €999/month<br>○ Between €1.000 and 1.499/month<br>○ Between €1.500 and 1.999/month<br>○ Between €2.000 and 2.499/month<br>○ Between €2.500 and 2.999/month<br>○ Between €3.000 and 3.499/month<br>○ Between €3.500 and 3.999/month<br>○ Between €4.000 and 4.999/month<br>○ Between €5.000 and 5.999/month<br>○ Between €6.000 and 6.999/month<br>○ Between €7.000 and 7.999/month<br>○ Between €8.000 and 8.999/month<br>○ Between €9.000 and 9.999/month<br>○ More than €10.000/month<br>○ I don't know, I do not want to tell this
1.10	Do you, father, have an unpaid job (except for caregiving)?	○ Yes<br>○ No
1.10.1	***If 'Do you, father, have an unpaid job (except for caregiving)?' is equal to 'Yes' answer this question:***<br>Father: if yes, for how many hours per month?	
1.11	Did you hire extra baby-sitting because of the hospital admission of your child?	○ Yes<br>○ No
1.12	Did you partner, in the past week, work less than expected in order for him tot take care of you and/or other children because of the admission?	○ Yes<br>○ No
1.12.1	***If 'Did you partner, in the past week, work less than expected in order for him tot take care of you and/or other children because of the admission?' is equal to 'Yes' answer this question:***<br>If yes, how many hours less than expected?	

1.13	For how many days was your baby admitted to the hospital?	◯ 2.5 days<br>◯ 3 days<br>◯ 3.5 days<br>◯ 4 days<br>◯ 4.5 days<br>◯ 5 days<br>◯ 5.5 days<br>◯ 6 days<br>◯ 6.5 days<br>◯ 7 days<br>◯ 7.5 days<br>◯ 8 days<br>◯ > 8 days
1.14	How many times did your baby receive a new IV canula?	◯ Not, because he/she was treated with antibiotic suspension<br>◯ 1 x<br>◯ 2 x<br>◯ 3 x<br>◯ 4 x<br>◯ More often<br>◯ I do not remember<br>◯ My child only kept the first inserted canula for the whole treatment
1.15	How high were your travel expenses each day for travelling from your house to the hospital?	[ ] euro
1.16	Did you visit another doctor this week for your baby?	◯ Yes<br>◯ No
1.16.1	***If 'Did you visit another doctor this week for your baby?' is equal to 'Yes' answer this question:***<br>If yes, what kind of doctor?	[ ]
1.17	Did your baby experience fever this week?	◯ Yes<br>◯ No
1.17.1	***If 'Did your baby experience fever this week?' is equal to 'Yes' answer this question:***<br>If yes, how high was the fever?	[ ] degrees Celcius

1.18	Did your child experience any side effects of the antibiotic therapy?	Vomiting<br>Change is defecation pattern<br>Rash<br>Other<br>None
1.19	What kind of feeding do you give to your baby?	Breastmilk<br>Formula milk<br>Combination of breast- and formulamilk
1.20	How does your child sleep?	Goes to sleep easily and wakes up satisfied<br>Going to sleep is sometimes difficult, but does eventually sleep well<br>Going to sleep is difficult, cries often, hard to comfort
1.21	Did your baby receive any other medication (besides vitamins)?	Yes<br>No
1.21.1	***If 'Did your baby receive any other medication (besides vitamins)?' is equal to 'Yes' answer this question:***<br>If yes, which medication?	

ENGLISH: Survey day 7 - Outro

Thank you for helping us by filling in this survey. You will receive the second survey in two weeks. Kind regards, RAIN team

Survey 'ENGLISH survey day 21'

ENGLISH survey day 21 - Introduction

Dear parents/guardians, We have asked you permission to send two surveys as follow up on the RAIN study. This is the second survey, which has to be filled in around 3 weeks after initiation of therapy. Thank you for your help! RAIN team

ENGLISH survey day 21 - ENGLISH survey day 21

Number	Question	Answers
1.1	Did you visit another doctor this week for your baby?	○ Yes<br>○ No
1.1.1	***If 'Did you visit another doctor this week for your baby?' is equal to 'Yes' answer this question:***<br>If yes, what kind of doctor?	[ ]
1.2	Did your baby experience fever this week?	○ Yes<br>○ No
1.2.1	***If 'Did your baby experience fever this week?' is equal to 'Yes' answer this question:***<br>If yes, how high was the fever?	[ ] degrees Celcius
1.3	What kind of feeding do you give to your baby?	○ Breastmilk<br>○ Formula milk<br>○ Combination of breast- and formulamilk
1.4	How does your child sleep?	○ Goes to sleep easily and wakes up satisfied<br>○ Going to sleep is sometimes difficult, but does eventually sleep well<br>○ Going to sleep is difficult, cries often, hard to comfort
1.5	Did your baby receive any other medication (besides vitamins)?	○ Yes<br>○ No
1.5.1	***If 'Did your baby receive any other medication (besides vitamins)?' is equal to 'Yes' answer this question:***<br>If yes, which medication?	[ ]

ENGLISH survey day 21 - Outro

Thank you for helping us by filling in this survey. This was the last survey of the RAIN study. Kind regards, RAIN team

Survey 'ENGLISH: microbiome T = 6 months'

ENGLISH: microbiome T = 6 months - Introduction

Dear parents/guardians, You have agreed to collect fecal samples in the first year of life of your child. It is time to collect a sample for the 4th time. 1. Please collect some stool from the diaper of your baby in the tube with the label: RAIN FECES 4 T = 6 months and put it directly back into the freezer. 2. Please answer the following questions.

ENGLISH: microbiome T = 6 months - questionnaire microbiome T = 6 months

Number	Question	Answers
1.1	What kind of feeding do you give to your child?	○ Breastmilk<br>○ Formula milk<br>○ Combination of breast- and formulamilk
1.2	Did you child start with solid food yet?	○ Yes<br>○ No
1.3	Are there older children living with you and your child in the same house?	○ no<br>○ yes, 1<br>○ yes, 2<br>○ yes, 3<br>○ yes, more than 3
1.3.1	***If 'Are there older children living with you and your child in the same house?' is not equal to 'no' answer this question:***<br>If yes, what age group are they in?	☐ < 4 years old<br>☐ 4-12 years old<br>☐ >12 years old
1.3.1.1	***If 'If yes, what age group are they in?' is equal to '< 4 years old' answer this question:***<br>Are your children, younger than 4 years old, going to daycare?	○ No<br>○ Yes, 1 day /week<br>○ Yes, 2 days /week<br>○ Yes, more than 2 days/week

1.4	Did your child experience any episodes of illness in the last 3 months?	☐ No<br>☐ Yes, nasal congestion/stuffy nose<br>☐ Yes, coughing<br>☐ Yes, fever > 38.0 degrees celcius<br>☐ Yes, diarrhea<br>☐ Yes, skin rash for more than 1 day
1.4.1	***If 'Did your child experience any episodes of illness in the last 3 months?' is not equal to 'No' answer this question:***<br>If yes, did you visit a doctor?	○ Yes<br>○ No
1.4.1.1	***If 'If yes, did you visit a doctor?' is equal to 'Yes' answer this question:***<br>If yes, please specify who (example: general practitioner, pediatrician)	
1.5	Has your child been readmitted to the hospital in the last 3 months?	○ Yes<br>○ No
1.5.1	***If 'Has your child been readmitted to the hospital in the last 3 months?' is equal to 'Yes' answer this question:***<br>If yes, for what reason?	
1.6	Did your child receive another course of antibiotics in the past 3 months?	○ Yes<br>○ No
1.6.1	***If 'Did your child receive another course of antibiotics in the past 3 months?' is equal to 'Yes' answer this question:***<br>If you remember the name of the treatment, please write it down	

ENGLISH: microbiome T = 6 months - Outro

In 6 months you will receive the last questionnaire and collect the last stool sample. Thank you for your help! RAIN team
