# Supplementary material for: RAIN study: a protocol for a randomised controlled trial evaluating efficacy, safety and cost-effectiveness of intravenous-to-oral antibiotic switch therapy in neonates with a probable bacterial infection
Source: BMJ Open. 2019 Jul 9;9(7):e026688. doi: 10.1136/bmjopen-2018-026688 (PMC6615779; doi:10.1136/bmjopen-2018-026688)
Supplement: Author's manuscript [file bmjopen-2018-026688.draft_revisions.pdf]

BMJ Open is committed to open peer review. As part of this commitment we make the peer review history of every article we publish publicly available.

When an article is published we post the peer reviewers' comments and the authors' responses online. We also post the versions of the paper that were used during peer review. These are the versions that the peer review comments apply to.

The versions of the paper that follow are the versions that were submitted during the peer review process. They are not the versions of record or the final published versions. They should not be cited or distributed as the published version of this manuscript.

BMJ Open is an open access journal and the full, final, typeset and author-corrected version of record of the manuscript is available on our site with no access controls, subscription charges or pay-per-view fees (http://bmjopen.bmj.com).

If you have any questions on BMJ Open's open peer review process please email info.bmjopen@bmj.com

BMJ Open

Intravenous-to-oral antibiotic switch therapy for suspected neonatal bacterial infections: a randomized controlled trial to study clinical efficacy, safety, and cost-effectiveness

RAIN study: Reduction of intravenous Antibiotics In Neonates

Journal:	*BMJ Open*
Manuscript ID	bmjopen-2018-026688
Article Type:	Protocol
Date Submitted by the Author:	14-Sep-2018

Complete List of Authors:	Keij, Fleur; Erasmus MC Sophia, Pediatrics, Division of Neonatology<br>Kornelisse, René; Erasmus MC Sophia, Pediatrics, Division of Neonatology<br>Hartwig, Nico; Sint Franciscus Vlietland Groep, Pediatrics<br>Mauff, Katya; Erasmus MC, Biostatistics<br>Poley, Marten; Erasmus MC Sophia, Pediatric Surgery; Erasmus Universiteit Rotterdam, Medical Technology Assessment (iMTA)<br>Allegaert, Karel; Erasmus MC Sophia, Pediatrics, Division of Neonatology<br>Reiss, Irwin; Erasmus MC Sophia, Pediatrics, Division of Neonatology<br>Tramper-Stranders, Gerdien; Erasmus MC Sophia, Pediatrics, Division of Neonatology; Sint Franciscus Vlietland Groep, Pediatrics

Keywords:	amoxicillin-clavulanic acid, antibiotic stewardship, neonatal sepsis, intravenous-to-oral antibiotic switch therapy, cost-effectiveness, microbiome

SCHOLARONE™
Manuscripts

Intravenous-to-oral antibiotic switch therapy for suspected neonatal bacterial infections: a randomized controlled trial to study clinical efficacy, safety, and cost-effectiveness

RAIN study: Reduction of intravenous Antibiotics In Neonates

Fleur M. Keij (MD)[1], René F. Kornelisse (PhD)[1], Nico G. Hartwig (PhD)[2], K. Mauff (Msc)[3], Marten J. Poley (PhD)[4,5], Karel Allegaert (PhD)[1], Irwin K. M. Reiss (PhD)[1], Gerdien A. Tramper-Stranders (PhD)[1,2]

[1]Department of Pediatrics, Division of Neonatology, Erasmus MC- Sophia Children's Hospital, Rotterdam, The Netherlands
[2]Department of Pediatrics, Franciscus Gasthuis & Vlietland, Rotterdam, The Netherlands
[3]Department of Biostatistics, Erasmus MC - University Medical Centre, Rotterdam, The Netherlands
[4] Intensive care and Department of Pediatric Surgery, Erasmus MC - Sophia Children's Hospital, Rotterdam, The Netherlands
[5] Institute for Medical Technology Assessment (iMTA), Erasmus University Rotterdam, Rotterdam, The Netherlands

Correspondence to:

Fleur M. Keij (MD)

Department of Pediatrics, Division of Neonatology, Erasmus MC- Sophia Children Hospital

3015 CN Rotterdam, The Netherlands

f.keij@erasmusmc.nl

Wordcount: abstract 255, manuscript 3239

For peer review only

For peer review only - http://bmjopen.bmj.com/site/about/guidelines.xhtml

1. Abstract

Introduction: High morbidity and mortality rates of proven bacterial infection are the main reason for substantial use of intravenous antibiotics in neonates during the first week of life. In older children, intravenous-to oral switch after 48 hours of intravenous therapy has been shown to have many advantages and is nowadays commonly practiced. We therefore aim to evaluate the effectiveness, safety, and cost-effectiveness of an early intravenous to oral switch in neonates with a probable or culture-negative bacterial infection.

Methods and analysis: We present a protocol for a multicenter randomised trial assessing the non-inferiority of an early intravenous- to oral antibiotic switch compared to a full course of intravenous antibiotics in neonates (0-28 days of age) with a probable bacterial infection.

Five hundred-and fifty patients will be recruited in 17 hospitals in the Netherlands. After 48 hours of intravenous treatment, they will be assigned to either continue for another 5 days with intravenous therapy (control) or switch to amoxicillin/clavulanic acid suspension for 5 days (intervention). Both groups will be treated for a total of 7 days. The primary outcome will be bacterial reinfection within 28 days after treatment. Secondary outcomes are the pharmacokinetic profile of oral amoxicillin/clavulanic acid, the impact of an early switch on quality of life, the cost-effectiveness of oral therapy, and the microbiome development.

Ethics and dissemination: This study is approved by the Medical Ethics Committee of the Erasmus Medical Center. Results will be presented in peer-reviewed journals and at international conferences.

Registration: The trial has been registered in Clinicaltrials.gov Trial number NCT03247920.

For peer review only - http://bmjopen.bmj.com/site/about/guidelines.xhtml

2. Strengths and limitations of the study

Max five short bullet points, no longer than one sentence each, that relate specifically **to the methods.**

- Important clinical question regarding neonatal care worldwide
- Non-inferiority study design
- The results of this study can be included in antibiotic stewardship programs
- Only probable infections are included in this study; low reinfection rate to be expected

For peer review only

For peer review only - http://bmjopen.bmj.com/site/about/guidelines.xhtml

3. Introduction

Antibiotics are among the most prescribed drugs in the pediatric population. With increasing concerns regarding microbial resistance leading to potential multi-drug resistant infections, antibiotic stewardship programs (ASPs) have been developed. The main goal of these programs is optimization of the use of antibiotics. Moreover, unnecessary use should be reduced since antibiotics do interfere with the development of the gut microbiome in early stages of life, influencing the risk of developing several diseases such as asthma, diabetes and obesity later in life (1-3). Initially started in adult population, these programs have since been implemented in the pediatric field, starting with the formation of the Pediatric Committee on Antimicrobial Stewardship (AS) by the Pediatric Infectious Diseases Society (PIDS) in 2010. One of the principles of AS is the appropriate and timely de-escalation of antibiotic use including a switch from parenteral to oral therapy in clinically well children. An early intravenous-to-oral switch leads to many advantages such as a reduction in hospital stay and associated health costs and is currently described in several guidelines. (4)

However, oral switch therapy is not yet widely practiced in neonates, who are at high risk for infections due to their immature immune system. When a bacterial infection is suspected, either based on maternal risk factors or subtle clinical symptoms, antibiotic therapy is immediately started. In the best-case scenario, symptoms disappear, inflammatory parameters are reassuring and cultures remain negative after which therapy can be stopped after 36-48 hours. In some of the neonates (1% of living births) the presence or absence of a bacterial infection cannot be established. They may have elevated inflammatory parameters or show clinical signs, although (blood) cultures remain sterile; so-called “probable bacterial infection” or “culture-negative neonatal infection”. (5) In such cases, antibiotic therapy is usually continued intravenously for, on average, seven days. (6)

For peer review only - http://bmjopen.bmj.com/site/about/guidelines.xhtml

In Low and Middle Income countries (LMIC), the above practice is not common due to limited resources. In order to reduce mortality following neonatal infections there is a need for good home-based antibiotic treatment which has led to several large randomized trials on the use of oral antibiotics. These studies showed that a simplified regimen with a switch to oral amoxicillin after 48 hours is as effective and safe as parenteral therapy. (7-9) Results of those studies have been incorporated in the WHO guidelines for the management of neonatal sepsis. (10) Unfortunately, this approach has not yet led to further research in a High Income Setting. A recent systematic review by our group provides an overview of all studies (since 1965) using oral antibiotics. (submitted) The main findings are that adequate antibiotic serum levels can be reached, although peak-levels are lower and appear later in time compared to parenteral administration. (11, 12) Small efficacy studies evaluating an early oral switch in neonates with a possible or proven infection are promising. No increase in reinfection rate was observed and the benefits of oral therapy included reduction of hospital stay and an increase in the number of exclusively breastfed newborns. (13, 14) Unfortunately, study sizes were small and the majority of studies were not randomized. Furthermore, no full cost-effectiveness analyses of pediatric intravenous-to-oral switch therapy have been reported to date. This represents an important knowledge gap, considering that, in the current era of austerity and rising health care costs, it is increasingly required to demonstrate whether or not new treatment strategies offer good value for money.

Together with the uncertainties about bioavailability, the lack of firm evidence delays further implementation in a high resource setting. When intravenous-to-oral antibiotic switch therapy in neonatal infections is proven safe and effective, it can lead to a great improvement in quality of life for both child and parents. Furthermore, it can reduce the length of hospital stay, which will have positive effects on rising health care costs.

For peer review only

Objectives

The primary objective is to demonstrate non-inferiority of intravenous-to-oral antibiotic switch therapy in clinically well neonates with a probable bacterial infection compared to a complete course of intravenous therapy. The secondary objectives are: 1) to describe pharmacokinetics of oral amoxicillin clavulanic acid in neonates; 2) to quantify the cost-effectiveness of oral antimicrobial switch therapy in neonates; 3) to evaluate the quality of life in relation to switch therapy in neonates; 4) to study modification of the gut microbiome in relation to type and administration of antibiotics and 5) to explore the value of new diagnostic strategies on bacterial infection using left over blood culture material that remains after culture-time has passed.

For peer review only

4. Methods and Analysis

Study design

The RAIN (**R**eduction of intravenous **A**ntibiotics **I**n **N**eonates) study is a multicenter, randomized controlled, open label, non-inferiority trial.

Patients will be recruited in 17 large teaching hospitals in the Netherlands.

For peer review only

Outcome measures

The primary outcome is bacterial (re)-infection within 28 days after finishing antimicrobial therapy, defined as clinical symptoms of bacterial infection and fever (> 38.0 °C) or hypothermia (< 36.0 °C) and elevated inflammatory parameters with a need for prolonged antimicrobial therapy (> 48h). Proven or suspected viral infection, for which no antibiotics are initiated or antibiotics are stopped within 48-72 hours (after return of culture and/or viral results) are not considered as a bacterial (re)infection.

Secondary outcomes are duration of hospitalization; total costs and cost effectiveness; clinical side effects of antibiotic therapy; pharmacokinetic profile of oral amoxicillin and clavulanic acid; impact on quality of life, and the additional yield of Next Generation Sequencing (NGS) as a diagnostic tool for neonatal sepsis and the gut microbiome development in the first year of life.

Participants

Neonates are eligible for participation if they have a postmenstrual age of 35 weeks or more, are 0-28 days old, have a body weight of two kilograms or more and have a “probable bacterial infection” or “culture-negative neonatal sepsis” with elevated inflammatory parameters for which the pediatrician has decided to complete a full course of intravenous antibiotics. All neonates with a proven bacterial infection, e. g. positive blood- or cerebrospinal fluid culture or a severe clinical sepsis requiring intensive care treatment are excluded from participation. In- and exclusion criteria are described in table 1.

For peer review only - http://bmjopen.bmj.com/site/about/guidelines.xhtml

Participants will be screened by the local pediatrician or investigator and randomized after 48 hours of intravenous treatment after informed consent has been obtained.

Intervention

The intervention group will switch to oral amoxicillin/clavulanic acid after at least 48 hours of intravenous antibiotics. The control group will receive the full course of intravenous antibiotics, which consists of penicillin and gentamicin in case of early onset sepsis in most participating centers. Both groups will be treated for a total of 7 days. The trial flow diagram is outlined in figure 1.

Dosage, method of administration

A daily oral dose of 75/18.75 mg/kg of amoxicillin/clavulanic acid, thus 25/6.25 mg/kg every 8 hours, will be administered. The mode of administration is orally via suspension, or in case of feeding problems (with the exception of vomiting) per nasopharyngal tube. If the neonate vomits significantly within 30 minutes after administration, the administration will be repeated.

Randomization and blinding

Participants will be randomized after 48 hours of intravenous treatment by a web-based program according to a stratified block randomization (stratified per site in alternated blocks of 2 and 4). Randomization ratio will be 1:1. Patients and health care professionals will not be blinded, because of the nature of treatments (intravenous versus oral therapy); the primary investigators performing the statistical analysis will be blinded for the allocated treatment.

Follow up and outcome assessment

Neonates will only be discharged from the hospital if they have shown to tolerate the oral antibiotic

For peer review only - http://bmjopen.bmj.com/site/about/guidelines.xhtml

treatment for at least 2 doses and are clinically well. Parents will be interviewed by telephone on the first day after discharge and on day 14 after initiation of antibiotic therapy by the local study investigator or research nurse. The local investigator will assess signs of infections and side effects and events. In addition to that parents will receive a survey on day 7 and day 21 with questions regarding possible signs of infection, side effects, quality of life and cost effectiveness. The primary outcome of the study is estimated five weeks after initiation of antibiotic therapy, when the patient will be seen by the local investigator or research nurse for a final visit at the outpatient department. Follow up of neonates in the control group will be identical.

Power- and statistical analysis

Relapse of proven GBS infection is estimated to occur in 0-8% of patients, but may be caused by a different strain (15). Two epidemiological studies estimated the recurrence rate to be 1% in proven bacterial neonatal infections. (16, 17) The current study focuses on probable infections (in which the recurrence rate is likely even lower, although evidence for this is lacking); we therefore assume a 1 % relapse rate (including new non-related infections) in both groups. Based on a non-inferiority margin of 3% (minimum success rate 96 %), 2 x 231 patients are needed for a power of 90% using a 1-sided alpha of 0.025. Dropout or withdrawal is estimated to be approximately 5-10%. We will therefore include 2 x 275 neonates (total = 550).

A formal interim analysis will be performed after occurrence of the first four primary outcomes. The DSMB will assess the results and it will be determined whether the occurrence of the primary outcome was related to the index suspected bacterial infection or more likely to be an unrelated infection. The outcome of that interim analysis will determine after how many primary outcomes the next interim analysis will be performed. When ≥ 75% of related cases occur in the intervention arm in two

For peer review only - http://bmjopen.bmj.com/site/about/guidelines.xhtml

consecutive interim analyses, the study will be stopped prematurely.

Both per-protocol analysis and intention-to-treat analysis will be performed. A p-value <0.025 will be considered as significant. Regarding the primary outcome, 28-day cumulative bacterial infection rate; proportions will be defined and used to compare both treatment strategies. Subgroup analysis will be performed on late preterm neonates (GA 35-37 weeks). Further, mixed effect models will be used to take into account the multiple centers. The secondary outcomes will be analyzed using a t-test or Mann-Whitney U test and in the case of categorical data a Chi-square test will be used.

Pharmacokinetics

Blood samples of the first 30 patients treated with oral antibiotics (intervention arm) will be analyzed in order to confirm that plasma concentrations are within target range and to endorse safety and dosing of the investigated product. It is thought that time above MIC should be at least 40-50% in order for treatment with penicillins to be adequate. (18) Therefore, T>MIC should be at least 50% in 90% of the cases to be considered adequate, otherwise dosing needs to be adjusted. A target MIC of 8 mg/l is chosen for amoxicillin since this is the clinical breakpoint for *E. coli* susceptibility defined by the European Committee on Antimicrobial Susceptibility (Eucast). (19) In a later stage, sampling will be continued in order to develop a PK/PD model and to perform a population pharmacokinetic analysis. For the first 30 patients, two blood samples (0.5 cc whole blood) will be collected; the first sample 4-6 hours after the first or second dose, the second sample 6-8 hours after the second or third dose of interventional product. After the first analysis on samples from 30 patients; sampling will be continued through the collection of a single blood sample per patient. Samples will be handled within one hour after collection by the local laboratory. The sample will be centrifuged at 3000 rpm for ten minutes and the recovered plasma will be stored at -80°C. Once enough samples have been collected for analysis,

For peer review only - http://bmjopen.bmj.com/site/about/guidelines.xhtml

they will be shipped to the Erasmus MC for analysis. Serum concentrations of both amoxicillin and clavulanic acid will be measured. For each measurement 50 microliters of plasma is needed. Analysis will be performed batchwise combining both Liquid Chromatography (LC) and Mass Spectometry (MS). Analysis will be will be performed using nonlinear mixed effects modeling (NONMEM).

Storage of left over blood material

Left over material of blood culture before initiation of antibiotic treatment will be stored at -80°C. Molecular analysis will be performed using next generation sequencing (NGS) methods, evaluating the additional yield of those techniques on bacterial detection compared to blood culture. Analysis will be performed batchwise, meaning that the additional value will be determined afterwards and that results will not be immediately available for the treating clinician.

Gut microbiome

Microbiome alterations following oral amoxicillin/clavulanic acid will be evaluated in 80 participants. Fecal samples will be collected at 5 different moments in the first year of life (around day 7, 30, 90, 180 and day 365) from neonates born vaginally. Samples will be collected from the diaper and immediately stored at -20 °C in a special box placed in the home freezer. Parents will receive a notification at each sampling moment including a questionnaire regarding episodes of antibiotic treatment, type of feeding, daycare attendance and the presence of siblings at home. After 365 days, all samples will shipped to the Erasmus MC and stored at -80°C until analysis in a specialized facility using high throughput sequencing targeted at the bacterial 16S rRNA locus.

Cost-effectiveness analysis

Following a superiority design, the cost-effectiveness analysis (CEA) will make a comparison between the

For peer review only - http://bmjopen.bmj.com/site/about/guidelines.xhtml

intervention and the control group by identifying, measuring and valuing the costs and patient outcomes of both treatment strategies.

Following established methods for economic evaluations and costing studies in health care, both medical costs and non-medical costs (i.e., costs of productivity losses) will be analyzed for both study groups. (20, 21) Total medical costs over the 28-day follow-up period will be calculated, including costs of intravenous antibiotics, costs of oral antibiotics, laboratory costs and costs of hospitalization days. In regard to readmission costs, only costs of a readmission for a bacterial (re)infection, as defined in the outcome measures section, will be added to the analysis. Resource consumption will be derived from electronic hospital databases. Unit prices will be calculated using real economic cost prices or using standard cost prices for health economic evaluations. Unit prices will be multiplied by the quantities for each resource used, and then summed over the separate types of resource to give a total cost per patient. Then, productivity costs in the patients' parents/caregivers, consisting of productivity losses regarding both paid and unpaid work, will be analyzed. These costs will be measured using the iMTA Productivity Cost Questionnaire (iPCQ) (22), completed by the parents during the scheduled follow up as described above. The productivity costs will be valued following the friction cost method. Briefly, the valuation of paid work will be based on average hourly labor costs per paid employee, whereas that of unpaid work will be based on the replacement costs for household work. Finally, all costs will be summated for each individual patient.

Regarding the patient outcomes, the CEA will look at the number of bacterial (re)-infections within 28 days after finishing of antimicrobial therapy. Building on the data on costs and outcomes described above, a cost-effectiveness ratio will be calculated (the final end-point of the cost-effectiveness analysis). This ratio will be expressed as incremental costs per bacterial (re-)infection avoided.

For peer review only - http://bmjopen.bmj.com/site/about/guidelines.xhtml

Otherwise, the CEA will focus on dominance of one treatment over the other with respect to lower cost and greater effect. Analysis of uncertainty in the estimation of the incremental cost-effectiveness ratio is illustrated through a cost-effectiveness plane (via bootstrapping). Where relevant, sensitivity analysis will be performed to assess the robustness of the analysis to certain assumptions.

Quality of Life

To get an impression of the impact of an early intravenous-to-oral antibiotic switch on the quality of life (QoL) of both parents and their child, parents will be asked to fill in a questionnaire on day 7 and day 21. Since there is no validated QoL instrument for neonates < 1 month, we developed a questionnaire ourselves that collects information on total admission days and readmission rate, symptoms, side effects, amount of intravenous cannulas inserted during admission, sleep quality of the patient, number of medical visits and medication use in the first month, feeding and breastfeeding success rate and parents' satisfaction.

Data collection and management

Data will be collected by the local investigator at each side and stored anonymously in a GCP proof digital database (Castor EDC, Cewit B.V). Two independent monitors will perform source data verification and assess performance of study procedures at each site following a predefined schedule.

Safety consideration

All serious life events (SAE) will be reported to the principal investigator within 48 hours. The principal investigator will report the adverse events to the Medical Ethics Committee (METC) of the Erasmus Medical Center and the Central Committee on Research Involving Human Subjects (CCMO). All SAEs will be followed until they have abated, or until a stable situation has been reached. An independent data

For peer review only - http://bmjopen.bmj.com/site/about/guidelines.xhtml

and safety monitoring board (DSMB) will be installed consisting of an epidemiologist/statistician, consultant neonatologist and paediatric infectious diseases specialist. The DSMB will evaluate the progress of the trial and will examine safety parameters at yearly intervals and through the performance of interim analyses when necessary. The adverse events will be listed and discussed with the DSMB.

For peer review only

For peer review only - http://bmjopen.bmj.com/site/about/guidelines.xhtml

5. Ethics and dissemination

This study was approved by the Medical Ethics Committee of the Erasmus Medical Center (NL51888.078.16, June 2017). The study will be conducted according to the principles of the Declaration of Helsinki (7th revision, October 2013) and in accordance with the Medical Research Involving Human Subjects Act (WMO) and other guidelines, regulations and Acts such as Good Clinical Practice. The Dutch parent organisation "Vereniging van Ouders van Couveusebabies (VOC)" was involved in the conceptualization of the trial and has given their approval for the execution of this study.

The trial has been registered in Clinicaltrials.gov Trial number NCT03247920.

The results of the primary and secondary aims will be published in peer-reviewed journals and presented at international conferences.

For peer review only

For peer review only - http://bmjopen.bmj.com/site/about/guidelines.xhtml

6. Discussion/Conclusion

Prolonged hospitalization for intravenous therapy is still common practice in Western societies when neonatal bacterial infection cannot be ruled out despite negative cultures. In-hospital treatment has a high impact on both patient and parents since prolonged hospitalisation often leads to infant-parent separation. Initiatives to prevent separation through the reduction of admission days are of increasing interest. The additional value of other biomarkers besides CRP, such as procalcitonin, in order to rule out bacterial infection has been evaluated. A recent study showed that using procalcitonin values as guidance for antibiotic therapy in neonates with a suspected sepsis leads to a reduction in days on therapy and hospitalization. However, when the infection could not be ruled out, as is the case in infants with a probable infection, therapy was still continued intravenously for seven days. (23) We propose the first randomized controlled trial in a high resource setting with a non-inferiority study design in order to demonstrate that oral antibiotic switch therapy is as effective as intravenous treatment in neonates with a probable bacterial infection. Furthermore, we will be the first to perform PK/PD analysis on oral amoxicillin/clavulanic acid on a large sample size. Once proven that serum concentrations following oral administration are adequate, oral switch therapy could potentially be expanded to clinically well infants with a proven infection.

We assume that once effectiveness and safety have been shown, implementation of intravenous-to-oral-switch-therapy will reduce the risks of hospital-related complications such as nosocomial infections and lead to an earlier return to a normal family setting with improvement in quality of life and better mother-child bonding. Furthermore we expect that the cost reduction will be substantial.

For peer review only

For peer review only - http://bmjopen.bmj.com/site/about/guidelines.xhtml

Author's contribution

GTS conceived the original idea for this trial. FK, RK, KM, MP, NH, KA and IR were involved in further conception and trial development. FK wrote the first draft. FK, GTS and RK rewrote the article. GTS, RK, KM, MP, NH, KA and IR critically revised the article for important intellectual content. All authors contributed to and approved the final version of the manuscript.

Funding statement

This work is supported by The Netherlands Organisation for Health Research and Development (ZonMW) grant number 848015005 and the Innovatiefonds Zorgverzekeraars.

Competing interests

The authors declare that they have no competing interests.

For peer review only

For peer review only - http://bmjopen.bmj.com/site/about/guidelines.xhtml

References

1. Cox LM, Yamanishi S, Sohn J, Alekseyenko AV, Leung JM, Cho I, et al. Altering the intestinal microbiota during a critical developmental window has lasting metabolic consequences. Cell. 2014;158(4):705-21.
2. Gray LE, O'Hely M, Ranganathan S, Sly PD, Vuillermin P. The Maternal Diet, Gut Bacteria, and Bacterial Metabolites during Pregnancy Influence Offspring Asthma. Front Immunol. 2017;8:365.
3. Johnson CC, Ownby DR. The infant gut bacterial microbiota and risk of pediatric asthma and allergic diseases. Transl Res. 2017;179:60-70.
4. Hyun DY, Hersh AL, Namtu K, Palazzi DL, Maples HD, Newland JG, et al. Antimicrobial stewardship in pediatrics: how every pediatrician can be a steward. JAMA Pediatr. 2013;167(9):859-66.
5. Fjalstad JW, Stensvold HJ, Bergseng H, Simonsen GS, Salvesen B, Ronnestad AE, et al. Early-onset Sepsis and Antibiotic Exposure in Term Infants: A Nationwide Population-based Study in Norway. Pediatr Infect Dis J. 2016;35(1):1-6.
6. Neonatal infection (early onset): antibiotics for prevention and treatment [Internet]. 2012 [cited 28-08-2018]. Available from: https://www.nice.org.uk/guidance/cg149.
7. African Neonatal Sepsis Trial g, Tshefu A, Lokangaka A, Ngaima S, Engmann C, Esamai F, et al. Simplified antibiotic regimens compared with injectable procaine benzylpenicillin plus gentamicin for treatment of neonates and young infants with clinical signs of possible serious bacterial infection when referral is not possible: a randomised, open-label, equivalence trial. Lancet. 2015;385(9979):1767-76.
8. Mir F, Nisar I, Tikmani SS, Baloch B, Shakoor S, Jehan F, et al. Simplified antibiotic regimens for treatment of clinical severe infection in the outpatient setting when referral is not possible for young infants in Pakistan (Simplified Antibiotic Therapy Trial [SATT]): a randomised, open-label, equivalence trial. Lancet Global Health. 2017;5(2):e177-e85.
9. Baqui AH, Saha SK, Ahmed AS, Shahidullah M, Quasem I, Roth DE, et al. Safety and efficacy of alternative antibiotic regimens compared with 7 day injectable procaine benzylpenicillin and gentamicin for outpatient treatment of neonates and young infants with clinical signs of severe infection when referral is not possible: a randomised, open-label, equivalence trial. Lancet Glob Health. 2015;3(5):e279-87.
10. A. Fuchs JB, S, Mathur, M. Sharland, J. N. Van Den Anker. Antibiotic Use for Sepsis in Neonates and Children: 2016 Evidence Update. WHO-Reviews. 2016.
11. Mir F. Thesis: High dose oral amoxicillin attains pharmacokinetic efficacy endpoints in young infants (0-59 days) with suspected sepsis - a population pharmacokinetic pilot study. EMORY Electronic Theses and Dissertations. 2013.
12. Autret E, Laugier J, Marimbu J, Vaillant MC, Furet Y, Breteau M. Comparison between IV and oral route amoxicillin administration in neonatal bacterial colonization. ARCH FR PEDIATR. 1988;45(9):679-82.
13. Gras-Le Guen C, Boscher C, Godon N, Caillon J, Denis C, Nguyen JM, et al. Therapeutic amoxicillin levels achieved with oral administration in term neonates. Eur J Clin Pharmacol. 2007;63(7):657-62.
14. Manzoni P, Esposito S, Gallo E, Gastaldo L, Farina D, Principi N. Switch therapy in full-term neonates with presumed or proven bacterial infection. J Chemother. 2009;21(1):68-73.
15. Green PA, Singh KV, Murray BE, Baker CJ. Recurrent group B streptococcal infections in infants: clinical and microbiologic aspects. J Pediatr. 1994;125(6 Pt 1):931-8.
16. Zangwill KM, Schuchat A, Wenger JD. Group B streptococcal disease in the United States, 1990: report from a multistate active surveillance system. MMWR CDC Surveill Summ. 1992;41(6):25-32.
17. Ekelund K, Konradsen HB. Invasive group B streptococcal disease in infants: a 19-year nationwide study. Serotype distribution, incidence and recurrent infection. Epidemiol Infect. 2004;132(6):1083-90.
18. de Hoog M, Mouton JW, van den Anker JN. New dosing strategies for antibacterial agents in the neonate. Semin Fetal Neonatal Med. 2005;10(2):185-94.
19. EUCAST. European Committee on Antimicrobial Susceptibility Testing
Breakpoint tables for interpretation of MICs and zone diameters
Version 8.1. 2018.

For peer review only - http://bmjopen.bmj.com/site/about/guidelines.xhtml

20.Nederland Z. Guidelines for economic evaluations of health care. In: Institute NHC, editor.: National Health Care Institute; 2016.
21.Nederland Z. Manual for costing research. Methods and reference prices for economic evaluations in health care. Updated edition. National Health Care Institute. 2015.
22.Bouwmans C, Krol M, Severens H, Koopmanschap M, Brouwer W, Hakkaart-van Roijen L. The iMTA Productivity Cost Questionnaire: A Standardized Instrument for Measuring and Valuing Health-Related Productivity Losses. Value Health. 2015;18(6):753-8.
23.Stocker M, van Herk W, El Helou S, Dutta S, Fontana MS, Schuerman F, et al. Procalcitonin-guided decision making for duration of antibiotic therapy in neonates with suspected early-onset sepsis: a multicentre, randomised controlled trial (NeoPIns). Lancet. 2017;390(10097):871-81.

For peer review only

For peer review only - http://bmjopen.bmj.com/site/about/guidelines.xhtml

Inclusion Criteria	Exclusion Criteria
• Neonates, postmentrual age ≥ 35+0 weeks, postnatal age of 0-28 days, body weight ≥ 2 kg<br>• Probable bacterial infection defined as clinical symptoms and/or maternal risk factors *and* elevated inflammatory markers for which empiric broad-spectrum antibiotic treatment was initiated and needs to be continued for > 48 hours<br>• Clinically well<br>• Tolerate oral feeding without overt vomiting	• Proven bloodstream infection<br>• Absence of blood culture<br>• Severe localized infection (meningitis, osteomyelitis, necrotizing enterocolitis)<br>• Severe clinical sepsis (compromised circulation, need for mechanical ventilation)<br>• Continuous need for a central venous line<br>• Severe hyperbilirubinemia exceeding the exchange level<br>• Parents inability to administer medication

Table 1: Inclusion and exclusion criteria RAIN study

For peer review only

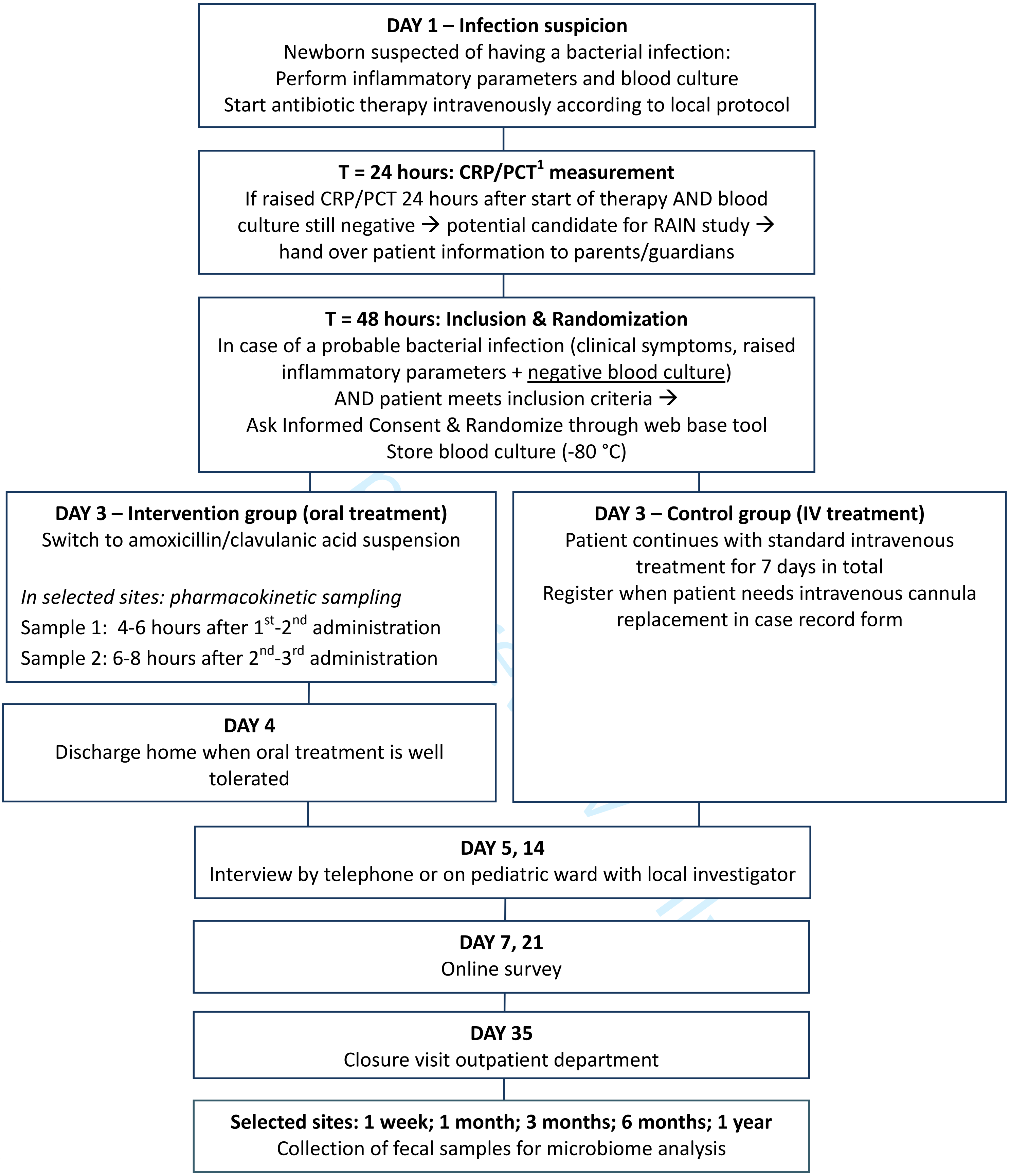

RAIN
Reduction of intravenous Antibiotics In Neonates

Figure 1: Flow chart of study procedures [1]CRP: C-reactive protein; PCT: procalcitonin; [2]IV: intravenous

BMJ Open

RAIN study: a protocol for a randomized controlled trial evaluating efficacy, safety and cost-effectiveness of intravenous-to-oral antibiotic switch therapy in neonates with a probable bacterial infection

Journal:	*BMJ Open*
Manuscript ID	bmjopen-2018-026688.R1
Article Type:	Protocol
Date Submitted by the Author:	28-Jan-2019

Complete List of Authors:	Keij, Fleur; Erasmus MC Sophia, Pediatrics, Division of Neonatology<br>Kornelisse, René; Erasmus MC Sophia, Pediatrics, Division of Neonatology<br>Hartwig, Nico; Sint Franciscus Vlietland Groep, Pediatrics<br>Mauff, Katya; Erasmus MC, Biostatistics<br>Poley, Marten; Erasmus MC Sophia, Pediatric Surgery; Erasmus Universiteit Rotterdam, Medical Technology Assessment (iMTA)<br>Allegaert, Karel; Erasmus MC Sophia, Pediatrics, Division of Neonatology<br>Reiss, Irwin; Erasmus MC Sophia, Pediatrics, Division of Neonatology<br>Tramper-Stranders, Gerdien; Erasmus MC Sophia, Pediatrics, Division of Neonatology; Sint Franciscus Vlietland Groep, Pediatrics

**Primary Subject Heading**:	Paediatrics
Secondary Subject Heading:	Infectious diseases
Keywords:	amoxicillin-clavulanic acid, intravenous-to-oral antibiotic switch therapy, cost-effectiveness, microbiome, neonatal infections

SCHOLARONE™ Manuscripts

RAIN study: a protocol for a randomized controlled trial evaluating efficacy, safety and cost-effectiveness of intravenous-to-oral antibiotic switch therapy in neonates with a probable bacterial infection

Fleur M. Keij (MD)[1], René F. Kornelisse (PhD)[1], Nico G. Hartwig (PhD)[2], K. Mauff (Msc)[3], Marten J. Poley (PhD)[4,5], Karel Allegaert (PhD)[1], Irwin K. M. Reiss (PhD)[1], Gerdien A. Tramper-Stranders (PhD)[1,2]

[1]Department of Pediatrics, Division of Neonatology, Erasmus MC- Sophia Children's Hospital, Rotterdam, The Netherlands
[2]Department of Pediatrics, Franciscus Gasthuis & Vlietland, Rotterdam, The Netherlands
[3]Department of Biostatistics, Erasmus MC - University Medical Centre, Rotterdam, The Netherlands
[4] Intensive care and Department of Pediatric Surgery, Erasmus MC - Sophia Children's Hospital, Rotterdam, The Netherlands
[5] Institute for Medical Technology Assessment (iMTA), Erasmus University Rotterdam, Rotterdam, The Netherlands

Correspondence to:

Fleur M. Keij (MD)

Department of Pediatrics, Division of Neonatology, Erasmus MC- Sophia Children Hospital

3015 CN Rotterdam, The Netherlands

f.keij@erasmusmc.nl

For peer review only

For peer review only - http://bmjopen.bmj.com/site/about/guidelines.xhtml

1. Abstract

Introduction: High morbidity and mortality rates of proven bacterial infection are the main reason for substantial use of intravenous antibiotics in neonates during the first week of life. In older children, intravenous-to oral switch after 48 hours of intravenous therapy has been shown to have many advantages and is nowadays commonly practiced. We therefore aim to evaluate the effectiveness, safety, and cost-effectiveness of an early intravenous to oral switch in neonates with a probable bacterial infection.

Methods and analysis: We present a protocol for a multicenter randomised trial assessing the non-inferiority of an early intravenous- to oral antibiotic switch compared to a full course of intravenous antibiotics in neonates (0-28 days of age) with a probable bacterial infection.

Five hundred-and fifty patients will be recruited in 17 hospitals in the Netherlands. After 48 hours of intravenous treatment, they will be assigned to either continue for another 5 days with intravenous therapy (control) or switch to amoxicillin/clavulanic acid suspension for 5 days (intervention). Both groups will be treated for a total of 7 days. The primary outcome will be bacterial (re-)infection within 28 days after treatment completion. Secondary outcomes are the pharmacokinetic profile of oral amoxicillin/clavulanic acid, the impact on quality of life, cost-effectiveness, impact on microbiome development and additional yield of molecular techniques in diagnosis of probable bacterial infection.

Ethics and dissemination: This study has been approved by the Medical Ethics Committee of the Erasmus Medical Center. Results will be presented in peer-reviewed journals and at international conferences.

Registration: The trial has been registered in Clinicaltrials.gov Trial number NCT03247920.

For peer review only - http://bmjopen.bmj.com/site/about/guidelines.xhtml

2. Strengths and limitations of the study

Max five short bullet points, no longer than one sentence each, that relate specifically **to the methods.**

- This study addresses an important clinical question regarding intravenous-to-oral antibiotic switch in neonatal care
- The results of this study can be included in antibiotic stewardship programs
- A non-inferiority study design is used
- The primary outcome assessment is challenging since it is impossible to state whether a new infection episode is a true re-infection since cultures of the first infection were sterile.
- Only probable bacterial infections are included in this study, thus results can not directly be applied to culture-proven infection.

For peer review only

For peer review only - http://bmjopen.bmj.com/site/about/guidelines.xhtml

3. Introduction

Antibiotics are among the most prescribed drugs in the pediatric population. With increasing concerns regarding microbial resistance leading to potential multi-drug resistant infections, antibiotic stewardship programs (ASPs) have been developed. The main goal of these programs is optimization of the use of antibiotics. Moreover, unnecessary use should be reduced since antibiotics do interfere with the development of the gut microbiome in early stages of life, influencing the risk of developing several diseases such as asthma, diabetes and obesity later in life (1-3). Initially started in adult population, these programs have, since then, been implemented in the pediatric field, starting with the formation of the Pediatric Committee on Antimicrobial Stewardship (AS) by the Pediatric Infectious Diseases Society (PIDS) in 2010. One of the principles of AS is the appropriate and timely de-escalation of antibiotic use including a switch from parenteral to oral therapy in clinically well children. An early intravenous-to-oral switch leads to many advantages such as a reduction in hospital stay and associated health costs and is currently described in several guidelines. (4)

However, oral switch therapy is not yet widely practiced in neonates. When a bacterial infection is suspected, either based on maternal risk factors or subtle clinical symptoms, antibiotic therapy is immediately started. In the best-case scenario, symptoms disappear, inflammatory parameters are reassuring and cultures remain negative after which therapy can be stopped after 36-48 hours. In some of the neonates (~1% of living births) the presence or absence of a bacterial infection cannot be established. (5) They may have elevated inflammatory parameters and show clinical signs, although (blood) cultures remain sterile; so-called “probable bacterial infection”. In such cases, antibiotic therapy is usually continued intravenously, up to seven days. (6)

For peer review only - http://bmjopen.bmj.com/site/about/guidelines.xhtml

In Low and Middle Income countries, the above practice is not common due to limited resources. In order to reduce mortality following neonatal infections there is a need for good home-based antibiotic treatment which has led to several large randomized trials on the use of oral antibiotics. These studies showed that a simplified regimen with a switch to oral amoxicillin after 48 hours is as effective and safe as parenteral therapy. (7-9) Results of those studies have been incorporated in the WHO guidelines for the management of neonatal sepsis. (10) Unfortunately, the outcomes cannot be applied to a higher income setting since the studies were based on clinical symptoms solely and no diagnostic tests were performed. This approach has not yet led to further research in High Income Settings.

Several small studies have been published on the use of different oral antibiotics, evaluating both pharmacokinetics and clinical efficacy. Regarding pharmacokinetics, studies have commonly found that adequate antibiotic serum levels can be reached following oral administration, although peak-levels are lower and appear later in time compared to parenteral administration. Furthermore a great inter individual variation was seen. (11, 12) Small efficacy studies evaluating an early oral switch in neonates with a possible or proven infection are promising. In a cohort study, 222 infants with possible and proven group B streptococcal (GBS) infection switched to oral amoxicillin in a high dosage after 48 hours of IV treatment. During the three-month follow up period, no reinfections occurred. (13) Similar results were found in a case-control study in which 36 infants with a possible or proven bacterial infection switched to oral cefpodoxime on day 3. No increase in reinfection rate was observed and the benefits of oral therapy included reduction of hospital stay and an increase in the number of exclusively breastfed newborns. (13, 14) Unfortunately, study sizes were small and studies were not randomized. Furthermore, no full cost-effectiveness analyses of pediatric intravenous-to-oral switch therapy have been reported to date. This represents an important knowledge gap, considering that, in the current era

For peer review only - http://bmjopen.bmj.com/site/about/guidelines.xhtml

of austerity and rising health care costs, it is increasingly required to demonstrate whether or not new treatment strategies offer good value for money.

Together with uncertainties about bioavailability after oral administration, the lack of firm evidence delays further implementation in a high resource setting. When intravenous-to-oral antibiotic switch therapy in neonatal infections is proven safe and effective, it can lead to a great improvement in quality of life for both child and parents. Furthermore, it can reduce the length of hospital stay, which will have positive effects on rising health care costs.

Objectives

The primary objective is to demonstrate non-inferiority of intravenous-to-oral antibiotic switch therapy in clinically well neonates with a probable bacterial infection compared to a complete course of intravenous therapy. The secondary objectives are: 1) to describe pharmacokinetics of oral amoxicillin clavulanic acid in neonates; 2) to quantify the cost-effectiveness of intravenous-to-oral switch therapy in neonates; 3) to evaluate the quality of life in relation to switch therapy in neonates; 4) to study modification of the gut microbiome in relation to type and administration of antibiotics and 5) to explore the additional value of molecular diagnostics on bacterial infection using left over blood culture material that remains after culture-time has passed.

For peer review only - http://bmjopen.bmj.com/site/about/guidelines.xhtml

4. Methods and Analysis

Study design

The RAIN (**R**eduction of intravenous **A**ntibiotics **I**n **N**eonates) study is a multicenter, randomized controlled, open label, non-inferiority trial. Patients will be recruited in 17 large teaching hospitals in the Netherlands. For details on participating hospitals we refer to the supplementary material.

Participants

Neonates are eligible for participation if they have a postmenstrual age of 35 weeks or more, are 0-28 days old, have a body weight of at least two kilograms and have a "probable bacterial infection" with elevated inflammatory parameters for which the pediatrician has decided to complete a full course of intravenous antibiotics. All neonates with a proven bacterial infection, e. g. positive blood- or cerebrospinal fluid culture or a severe clinical sepsis requiring intensive care treatment are excluded from participation. In- and exclusion criteria are described in table 1. Participants will be screened by the local pediatrician or investigator and randomized after 48 hours of intravenous treatment after informed consent has been obtained.

Inclusion Criteria	Exclusion Criteria
• Neonates, postmentrual age ≥ 35+0 weeks, postnatal age of 0-28 days, body weight ≥ 2 kg<br>• Probable bacterial infection defined as clinical symptoms and/or maternal risk factors *and* elevated inflammatory markers for which empiric broad-spectrum antibiotic treatment was initiated and needs to be continued for > 48 hours<br>• Clinically well<br>• Tolerate oral feeding without overt vomiting	• Proven bloodstream infection<br>• Absence of blood culture<br>• Severe localized infection (meningitis, osteomyelitis, necrotizing enterocolitis)<br>• Severe clinical sepsis (compromised circulation, need for mechanical ventilation)<br>• Continuous need for a central venous line<br>• Severe hyperbilirubinemia exceeding the exchange level<br>• Parents' inability to administer medication

Table 1: Inclusion and exclusion criteria RAIN study

For peer review only - http://bmjopen.bmj.com/site/about/guidelines.xhtml

Intervention

The intervention group will switch to oral amoxicillin/clavulanic acid after at least 48 hours of intravenous antibiotics. The control group will receive the full course of intravenous antibiotics, which consists of penicillin and gentamicin in case of early onset sepsis in most participating centers. Both groups will be treated for a total of 7 days. The trial flow diagram is outlined in figure 1.

Dosage, method of administration

A daily oral dose of 75/18.75 mg/kg of amoxicillin/clavulanic acid, thus 25/6.25 mg/kg every 8 hours, will be administered. The mode of administration is orally via suspension, or in case of feeding problems (with the exception of vomiting) per nasopharyngal tube. If the neonate vomits significantly within 30 minutes after administration, the administration will be repeated.

Randomization and blinding

Participants will be randomized after 48 hours of intravenous treatment by a web-based program according to a stratified block randomization (stratified per site in alternated blocks of 2 and 4). Randomization ratio will be 1:1. Randomization can under no circumstances be influenced by the study team. If parents want their child to be in a specific group they will be excluded from participation. Patients and health care professionals will not be blinded, because of the nature of treatments (intravenous versus oral therapy); the primary investigators performing the statistical analysis will be blinded for the allocated treatment.

Follow up

Neonates will only be discharged from the hospital if they have shown to tolerate the oral antibiotic treatment for at least 2 doses and are clinically well. Parents will be interviewed by telephone on the first day after discharge and on day 14 after initiation of antibiotic therapy by the local study investigator

For peer review only - http://bmjopen.bmj.com/site/about/guidelines.xhtml

For peer review only

or research nurse. The local investigator will assess antibiotic intake, signs of infection, side effects and events. In addition, parents will receive a survey on day 7 and day 21 with questions regarding possible signs of infection, side effects, quality of life and cost effectiveness. All surveys are available as supplementary material. The primary outcome of the study is assessed 28 days after treatment completion, when the patient will be seen by the local investigator or research nurse for a final visit at the outpatient department. Parents of participants in the oral treatment group will be asked to return the bottle with remaining antibiotic suspension in order to evaluate medication adherence through measurement of the remaining amount of medication present in the bottle. Follow up of neonates in the control group will be identical.

Power calculation

Relapse of proven Group B streptococcus (GBS) infection is estimated to occur in 0-8% of patients, but may be caused by a different strain. (15) Two epidemiological studies estimated the recurrence rate to be 1% in proven bacterial neonatal infections. (16, 17) The current study focuses on probable infections (in which the recurrence rate is likely even lower, although evidence for this is lacking). The incidence of community-acquired late onset sepsis in otherwise healthy neonates is low (~0.28 per 1000 livebirths).(18) Therefore, we assume a 1 % infection rate (including new non-related infections) in both groups. Based on a non-inferiority margin of 3% (minimum success rate 96 %), 2 x 231 patients are needed for a power of 90% using a 1-sided alpha of 0.025. Because of the first 30 patients undergoing PK measurements and uncertainty about the PK values, and potential dropout or withdrawal (estimated approximately 5-10%), we will include 2 x 275 neonates (total = 550). A post-hoc report on the design effect will be included.

For peer review only - http://bmjopen.bmj.com/site/about/guidelines.xhtml

Data collection and management

Data will be collected by the local investigator at each site and stored anonymously in a GCP proof digital database (Castor EDC, Cewit B.V). Two independent monitors will perform source data verification and assess performance of study procedures at each site following a predefined schedule.

Outcome measures and statistical analysis

Primary outcome

The primary outcome is bacterial (re-) infection within 28 days after finishing antimicrobial therapy, defined as clinical symptoms of infection and fever (> 38.0 °C) or hypothermia (< 36.0 °C) and elevated inflammatory parameters with a need for prolonged antimicrobial therapy for more than 48-72 hours. Proven or suspected viral infection, for which no antibiotics are initiated or antibiotics are stopped within 48-72 hours (after return of culture and/or viral results) are not considered as a bacterial (re-) infection. A chart will aid in decision making with respect to differentiation between bacterial infection and other causes. Both per-protocol analysis and intention-to-treat analysis will be performed. A p-value <0.025 will be considered as significant. Regarding the primary outcome, 28-day cumulative bacterial infection rate; proportions will be defined and used to compare both treatment strategies. Subgroup analysis will be performed on late preterm neonates (GA 35-37 weeks). Further, mixed effect models will be used to take into account the multiple centers.

Interim analysis

A formal interim analysis will be performed after occurrence of the first four primary outcomes. The Data Safety Monitoring Board (DSMB) will assess the results and it will be determined whether the occurrence of the primary outcome was related to the index suspected bacterial infection or more likely to be an unrelated infection. The outcome of that interim analysis will determine after how many

For peer review only - http://bmjopen.bmj.com/site/about/guidelines.xhtml

primary outcomes the next interim analysis will be performed. When more than 75% of related cases occur in the intervention arm in two consecutive interim analyses, the study will be stopped prematurely.

Secondary outcomes and their statistical analyses are described below.

Secundary outcomes

Pharmacokinetics

Blood samples of the first 30 patients treated with oral antibiotics (intervention arm) will be analyzed in order to confirm that plasma concentrations are within target range and to endorse safety and dosing of the investigated product. It is thought that time above MIC should be at least 40-50% in order for treatment with penicillins to be adequate. (19) Therefore, T>MIC should be at least 50% in 90% of the cases to be considered adequate, otherwise dosing needs to be adjusted. A target MIC of 8 mg/l is chosen for amoxicillin since this is the clinical breakpoint for *E. coli* susceptibility defined by the European Committee on Antimicrobial Susceptibility (Eucast). (20) In a later stage, sampling will be continued in order to develop a PK/PD model and to perform a population pharmacokinetic analysis. For the first 30 patients, two blood samples (0.5 ml whole blood) will be collected; the first sample 4-6 hours after the first or second dose, the second sample 6-8 hours after the second or third dose of oral suspension. In a subset of patients, we will collect 2 samples following the same administration in order to calculate an area under the curve (AUC). After the first analysis on samples from 30 patients; sampling will be continued through the collection of a single blood sample per patient. Samples will be handled within one hour after collection by the local laboratory. The sample will be centrifuged at 3000 rpm for ten minutes and the recovered plasma will be stored at -80°C. Once enough samples have been collected for analysis, they will be shipped to the Erasmus MC for analysis. Serum concentrations of both

For peer review only - http://bmjopen.bmj.com/site/about/guidelines.xhtml

amoxicillin and clavulanic acid will be measured. For each measurement 50 microliters of plasma is needed. Analysis will be performed batchwise combining both Liquid Chromatography (LC) and Mass Spectometry (MS). Analysis will be performed by descriptive statistics and by using nonlinear mixed effects modeling (NONMEM) including variables such as timing of blood drawing, timing of last meal, patient weight, center.

Molecular techniques for bacterial detection

Left over material of blood culture before initiation of antibiotic treatment will be stored at -80°C. Molecular analysis will be performed using next generation sequencing (NGS) methods, evaluating the additional yield of those techniques on bacterial detection compared to blood culture. Analysis will be performed batchwise, meaning that the additional value will be determined afterwards and that results will not be immediately available for the treating clinician nor for the DSMB in case of a (re-)infection. However results will be available at the end of the study and will be analysed in regard to primary outcomes that have occurred during the course of the study. The analysis of the Next Gen Sequencing (NGS) will initially be exploratory. If substantial differences are noted in the yield of recovered organisms from NGS compared to the blood culture, further analysis may be undertaken, exploring the relationship with clinical data such as inflammatory parameters and clinical illness. In that instance, mixed models will be used, and adjustments will be made for center.

Gut microbiome

Microbiome alterations following antibiotic therapy, both in the intervention and control group, will be evaluated in 80 participants from four centers. Fecal samples will be collected at 5 different moments in the first year of life (around day 7, 30, 90, 180 and day 365) from neonates born vaginally. Samples will be collected from the diaper and immediately stored at -20 °C in a special box placed in the home

For peer review only - http://bmjopen.bmj.com/site/about/guidelines.xhtml

freezer. Parents will receive a notification at each sampling moment including a questionnaire regarding episodes of antibiotic treatment, type of feeding, daycare attendance and the presence of siblings at home. After 365 days, all samples will shipped to the central research locations and stored at -80°C until analysis in a specialized facility using high throughput sequencing targeted at the bacterial 16S rRNA locus. Alpha diversity of all samples will be calculated by the Shannon index. We will identify time points in which the alpha diversity and relative abundances in operational taxonomical units differ between the infants with iv-treatment and infants with iv-oral switch with a smoothing spline analysis of variance (SS-ANOVA). Moreover, differences in the relative abundances of the genera will be tested with a linear mixed effect model with correction for the variables of other antibiotic use, feeding type, day care attendance/sibling and center.

Cost-effectiveness analysis

Following a superiority design, the cost-effectiveness analysis (CEA) will make a comparison between the intervention and the control group by identifying, measuring and valuing the costs and patient outcomes of both treatment strategies.

Following established methods for economic evaluations and costing studies in health care, both medical costs and non-medical costs (i.e., costs of productivity losses) will be analyzed for both study groups. (21, 22) Total medical costs over the 28-day follow-up period will be calculated, including costs of intravenous antibiotics, costs of oral antibiotics, laboratory costs and costs of hospitalization days. In regard to readmission costs, only costs of a readmission for a bacterial (re-) infection, as defined in the outcome measures section, will be added to the analysis. Resource consumption will be derived from electronic hospital databases. Unit prices will be calculated using real economic cost prices or using standard cost prices for health economic evaluations. Unit prices will be multiplied by the quantities for

For peer review only - http://bmjopen.bmj.com/site/about/guidelines.xhtml

each resource used, and then summed over the separate types of resource to give a total cost per patient. Then, productivity costs in the patients' parents/caregivers, consisting of productivity losses regarding both paid and unpaid work, will be analyzed (ignoring prearranged, regular maternity and paternity leave). These costs will be measured using the institute of Medical Technology Assessment (iMTA) Productivity Cost Questionnaire (iPCQ) (23), completed by the parents during the scheduled follow up as described above. The productivity costs will be valued following the friction cost method. Briefly, the valuation of paid work will be based on average hourly labor costs per paid employee, whereas that of unpaid work will be based on the replacement costs for household work. Finally, all costs will be summated for each individual patient.

Regarding the patient outcomes, the CEA will look at the number of bacterial (re-) infections within 28 days after finishing of antimicrobial therapy. Building on the data on costs and outcomes described above, a cost-effectiveness ratio will be calculated (the final end-point of the cost-effectiveness analysis). This ratio will be expressed as incremental costs per bacterial (re-)infection avoided. Otherwise, the CEA will focus on dominance of one treatment over the other with respect to lower cost and greater effect. Analysis of uncertainty in the estimation of the incremental cost-effectiveness ratio is illustrated through a cost-effectiveness plane (via bootstrapping). Where relevant, sensitivity analysis will be performed to assess the robustness of the analysis to certain assumptions.

Quality of life

To get an impression of the impact of an early intravenous-to-oral antibiotic switch on the quality of life (QoL) of both parents and their child, parents will be asked to fill in a questionnaire on day 7 and day 21. Since there is no validated QoL instrument for neonates < 1 month, we developed a questionnaire ourselves that collects information on total admission days and readmission rate, symptoms, side

For peer review only - http://bmjopen.bmj.com/site/about/guidelines.xhtml

effects, amount of intravenous cannulas inserted during admission, sleep quality of the patient, number of medical visits and medication use in the first month, feeding and breastfeeding success rate and parents' satisfaction. Outcome reporting will be mainly descriptive, if a trend is observed for some of the questions, mixed effect models can be used to further explore the effect of treatment and center.

Safety consideration

Serious life events (SAE) will be reported to the principal investigator within 48 hours. The principal investigator will report the adverse events to the Medical Ethics Committee (METC) of the Erasmus Medical Center and the Central Committee on Research Involving Human Subjects (CCMO). All SAEs will be followed until they have abated, or until a stable situation has been reached. An independent DSMB will be installed consisting of an epidemiologist/statistician, consultant neonatologist and paediatric infectious diseases specialist. The DSMB will evaluate the progress of the trial and will examine safety parameters at yearly intervals and through the performance of interim analyses when necessary. The adverse events will be listed and discussed with the DSMB.

Patient and Public Involvement

The Dutch parent organisation "Vereniging van Ouders van Couveusekinderen (VOC)" was involved in the conceptualization of the trial and has given their approval for the execution of this study. Patients will be asked at inclusion if they want to be informed on the final study results by email. Furthermore, a summary of findings will be published on the website of the VOC and on our own website.

For peer review only - http://bmjopen.bmj.com/site/about/guidelines.xhtml

5. Ethics and dissemination

This study was approved by the Medical Ethics Committee of the Erasmus Medical Center (NL51888.078.16, June 2017). The study will be conducted according to the principles of the Declaration of Helsinki (7th revision, October 2013) and in accordance with the Medical Research Involving Human Subjects Act (WMO) and other guidelines, regulations and Acts such as Good Clinical Practice.

The trial has been registered in Clinicaltrials.gov Trial number NCT03247920. The results of the primary and secondary aims will be published in peer-reviewed journals and presented at international conferences, followed by a press release.

For peer review only

For peer review only - http://bmjopen.bmj.com/site/about/guidelines.xhtml

6. Discussion/Conclusion

Prolonged hospitalization for intravenous therapy is still common practice in high income societies when neonatal bacterial infection cannot be ruled out despite negative cultures. In-hospital treatment has a high impact on both patient and parents since prolonged hospitalisation often leads to infant-parent separation. Initiatives to prevent separation through the reduction of admission days are of increasing interest. The additional value of other biomarkers besides CRP, such as procalcitonin, in order to rule out bacterial infection has been evaluated. A recent study showed that using procalcitonin values as guidance for antibiotic therapy in neonates with a suspected bacterial infection leads to a reduction in days on therapy and hospitalization. However, when the infection could not be ruled out, as is the case in infants with a probable infection, therapy was still continued intravenously for seven days. (24) We propose the first randomized controlled trial in a high resource setting with a non-inferiority study design in order to demonstrate that oral antibiotic switch therapy is as effective as intravenous treatment in neonates with a probable bacterial infection. Furthermore, we will be the first to perform PK/PD analysis on oral amoxicillin/clavulanic acid on a large sample size. Once proven that serum concentrations following oral administration are adequate, and non-inferiority of oral switch has been proven, oral switch therapy could potentially be expanded to clinically well infants with a proven infection.

This study has some limitations, the most important limitation relating to the primary outcome assessment. As mentioned in the methods section, only neonates with a probable bacterial infection will be included. It is therefore impossible to state whether or not a second infection is a reinfection or that it is caused by a different pathogen. We therefore developed a list of recommended diagnostics that should be determined when a participant returns with signs of a possible infection. Those results can be

For peer review only

used for correct and uniform assessment of the event and will help differentiating between a true bacterial infection, a probable bacterial infection or a viral infection.

Another limitation of the study relates to the fact that pharmacokinetic blood sampling will be performed in the intervention group. As this procedure carries a small risk of infections, bias may be introduced into the study. On the other hand, patients in the control group are at risk for infection when replacement or new insertion of an intravenous cannula is needed. Both situations can lead to a possible bacterial infection. We think, however, that the infection risk is low when performed correctly including good hygiene measurements. Moreover, neonates in both groups are under antibiotic treatment which also covers *S. aureus*.

Finally, bias may occur because of the oral treatment being administered out of the hospital setting. Since this treatment is home-based, parents' compliance in administering medication may be suboptimal, and we might underestimate the effectiveness of the intervention. In an attempt to overcome this limitation, infants will only be discharged when parents have shown to adequately administer the antibiotic suspension, parents will be phoned 1-2 days after discharge to evaluate medication administration, and parents will be asked to return the bottles with remaining antibiotic suspension at the final visit (which allows us to estimate the volume of the suspension actually administered).

We assume that once effectiveness and safety have been shown, implementation of intravenous-to-oral-switch-therapy will reduce the risks of hospital-related complications such as nosocomial infections and lead to an earlier return to a normal family setting with improvement in quality of life and better mother-child bonding. Furthermore we expect that the cost reduction will be substantial.

For peer review only
For peer review only - http://bmjopen.bmj.com/site/about/guidelines.xhtml

Author's contribution

GTS conceived the original idea for this trial. FK, RK, KM, MP, NH, KA and IR were involved in further conception and trial development. FK wrote the first draft. FK, GTS and RK rewrote the article. GTS, RK, KM, MP, NH, KA and IR critically revised the article for important intellectual content. All authors contributed to and approved the final version of the manuscript.

Funding statement

This work is supported by The Netherlands Organisation for Health Research and Development (ZonMW) grant number 848015005 and the Innovatiefonds Zorgverzekeraars.

Acknowledgements

We thank The Dutch parent organisation "Vereniging van Ouders van Couveusekinderen (VOC)" for their help in the conceptualization of the trial.

Competing interests

The authors declare that they have no competing interests.

Figure legends

1: Table 1: Inclusion and exclusion criteria RAIN study

2: **Figure 1: Flow chart of study procedures** [1]CRP: C-reactive protein; PCT: procalcitonin; [2]IV: intravenous

For peer review only

References

1. Cox LM, Yamanishi S, Sohn J, Alekseyenko AV, Leung JM, Cho I, et al. Altering the intestinal microbiota during a critical developmental window has lasting metabolic consequences. Cell. 2014;158(4):705-21.
2. Gray LE, O'Hely M, Ranganathan S, Sly PD, Vuillermin P. The Maternal Diet, Gut Bacteria, and Bacterial Metabolites during Pregnancy Influence Offspring Asthma. Front Immunol. 2017;8:365.
3. Johnson CC, Ownby DR. The infant gut bacterial microbiota and risk of pediatric asthma and allergic diseases. Transl Res. 2017;179:60-70.
4. Hyun DY, Hersh AL, Namtu K, Palazzi DL, Maples HD, Newland JG, et al. Antimicrobial stewardship in pediatrics: how every pediatrician can be a steward. JAMA Pediatr. 2013;167(9):859-66.
5. Fjalstad JW, Stensvold HJ, Bergseng H, Simonsen GS, Salvesen B, Ronnestad AE, et al. Early-onset Sepsis and Antibiotic Exposure in Term Infants: A Nationwide Population-based Study in Norway. Pediatr Infect Dis J. 2016;35(1):1-6.
6. Neonatal infection (early onset): antibiotics for prevention and treatment [Internet]. 2012 [cited 28-08-2018]. Available from: https://www.nice.org.uk/guidance/cg149.
7. African Neonatal Sepsis Trial g, Tshefu A, Lokangaka A, Ngaima S, Engmann C, Esamai F, et al. Simplified antibiotic regimens compared with injectable procaine benzylpenicillin plus gentamicin for treatment of neonates and young infants with clinical signs of possible serious bacterial infection when referral is not possible: a randomised, open-label, equivalence trial. Lancet. 2015;385(9979):1767-76.
8. Mir F, Nisar I, Tikmani SS, Baloch B, Shakoor S, Jehan F, et al. Simplified antibiotic regimens for treatment of clinical severe infection in the outpatient setting when referral is not possible for young infants in Pakistan (Simplified Antibiotic Therapy Trial [SATT]): a randomised, open-label, equivalence trial. Lancet Global Health. 2017;5(2):e177-e85.
9. Baqui AH, Saha SK, Ahmed AS, Shahidullah M, Quasem I, Roth DE, et al. Safety and efficacy of alternative antibiotic regimens compared with 7 day injectable procaine benzylpenicillin and gentamicin for outpatient treatment of neonates and young infants with clinical signs of severe infection when referral is not possible: a randomised, open-label, equivalence trial. Lancet Glob Health. 2015;3(5):e279-87.
10. A. Fuchs JB, S, Mathur, M. Sharland, J. N. Van Den Anker. Antibiotic Use for Sepsis in Neonates and Children: 2016 Evidence Update. WHO-Reviews. 2016.
11. Mir F. Thesis: High dose oral amoxicillin attains pharmacokinetic efficacy endpoints in young infants (0-59 days) with suspected sepsis - a population pharmacokinetic pilot study. EMORY Electronic Theses and Dissertations. 2013.
12. Autret E, Laugier J, Marimbu J, Vaillant MC, Furet Y, Breteau M. Comparison between IV and oral route amoxicillin administration in neonatal bacterial colonization. ARCH FR PEDIATR. 1988;45(9):679-82.
13. Gras-Le Guen C, Boscher C, Godon N, Caillon J, Denis C, Nguyen JM, et al. Therapeutic amoxicillin levels achieved with oral administration in term neonates. Eur J Clin Pharmacol. 2007;63(7):657-62.
14. Manzoni P, Esposito S, Gallo E, Gastaldo L, Farina D, Principi N. Switch therapy in full-term neonates with presumed or proven bacterial infection. J Chemother. 2009;21(1):68-73.
15. Green PA, Singh KV, Murray BE, Baker CJ. Recurrent group B streptococcal infections in infants: clinical and microbiologic aspects. J Pediatr. 1994;125(6 Pt 1):931-8.
16. Zangwill KM, Schuchat A, Wenger JD. Group B streptococcal disease in the United States, 1990: report from a multistate active surveillance system. MMWR CDC Surveill Summ. 1992;41(6):25-32.
17. Ekelund K, Konradsen HB. Invasive group B streptococcal disease in infants: a 19-year nationwide study. Serotype distribution, incidence and recurrent infection. Epidemiol Infect. 2004;132(6):1083-90.
18. Giannoni E, Agyeman PKA, Stocker M, Posfay-Barbe KM, Heininger U, Spycher BD, et al. Neonatal Sepsis of Early Onset, and Hospital-Acquired and Community-Acquired Late Onset: A Prospective Population-Based Cohort Study. J Pediatr. 2018;201:106-14 e4.
19. de Hoog M, Mouton JW, van den Anker JN. New dosing strategies for antibacterial agents in the neonate. Semin Fetal Neonatal Med. 2005;10(2):185-94.
20. EUCAST. European Committee on Antimicrobial Susceptibility Testing

For peer review only - http://bmjopen.bmj.com/site/about/guidelines.xhtml

Breakpoint tables for interpretation of MICs and zone diameters
Version 8.1. 2018.
21. Nederland Z. Guidelines for economic evaluations of health care. In: Institute NHC, editor.: National Health Care Institute; 2016.
22. Nederland Z. Manual for costing research. Methods and reference prices for economic evaluations in health care. Updated edition. National Health Care Institute. 2015.
23. Bouwmans C, Krol M, Severens H, Koopmanschap M, Brouwer W, Hakkaart-van Roijen L. The iMTA Productivity Cost Questionnaire: A Standardized Instrument for Measuring and Valuing Health-Related Productivity Losses. Value Health. 2015;18(6):753-8.
24. Stocker M, van Herk W, El Helou S, Dutta S, Fontana MS, Schuerman F, et al. Procalcitonin-guided decision making for duration of antibiotic therapy in neonates with suspected early-onset sepsis: a multicentre, randomised controlled trial (NeoPIns). Lancet. 2017;390(10097):871-81.

For peer review only

DAY 1 – Infection suspicion
Newborn suspected of having a bacterial infection following risk assessment national protocol:
Perform inflammatory parameters and blood culture
Start antibiotic therapy intravenously according to national protocol

T = 24 hours: CRP/PCT[1] measurement
If raised CRP/PCT 24 hours after start of therapy AND blood culture still negative → potential candidate for RAIN study → hand over patient information to parents/guardians

T = 48 hours: Inclusion & Randomization
In case of a probable bacterial infection (risk factor and/or clinical symptoms at onset AND raised inflammatory parameters AND negative blood culture)
AND patient meets inclusion criteria →
Ask Informed Consent & Randomize through web base tool
Store blood culture (-80 °C)

DAY 3 – Intervention group (oral treatment)
Switch to amoxicillin/clavulanic acid suspension

In selected sites: pharmacokinetic sampling
Sample 1: 1st-2nd administration
Sample 2: 2nd-3rd administration

DAY 4
Discharge home when oral treatment is well tolerated

DAY 3 – Control group (IV treatment)
Patient continues with standard intravenous treatment for 7 days in total
Register when patient needs intravenous cannula replacement in case record form

DAY 5, 14
Interview by telephone or on pediatric ward with local investigator

DAY 7, 21
Online survey

DAY 35
Closure visit outpatient department

Selected sites: 1 week; 1 month; 3 months; 6 months; 1 year
Collection of fecal samples for microbiome analysis

RAIN
Reduction of intravenous Antibiotics In Neonates

Figure 1: Flow chart of study procedures [1]CRP: C-reactive protein; PCT: procalcitonin; [2]IV: intravenous

Supplementary material:

RAIN study: a protocol for a randomized controlled trial evaluating efficacy, safety and cost-effectiveness of intravenous-to-oral antibiotic switch therapy in neonates with a probable bacterial infection

1. SPIRIT checklist
2. Overview participating sites
3. Risk assessment tables for suspected early-onset bacterial infection from the Dutch national guideline: *NVK Richtlijn: Preventie en behandeling van early-onset neonatale infecties*
4. Questionnaires for assessment of cost-effectiveness (CEA) and Quality of Life (QoL).
5. Questionnaires for microbiome analysis

For peer review only

For peer review only - http://bmjopen.bmj.com/site/about/guidelines.xhtml

SPIRIT 2013 Checklist: Recommended items to address in a clinical trial protocol and related documents*

Section/item	Item No	Description	Page
**Administrative information**			
Title	1	Descriptive title identifying the study design, population, interventions, and, if applicable, trial acronym	1
Trial registration	2a	Trial identifier and registry name. If not yet registered, name of intended registry	2 & 15
	2b	All items from the World Health Organization Trial Registration Data Set	yes
Protocol version	3	Date and version identifier	yes
Funding	4	Sources and types of financial, material, and other support	18
Roles and responsibilities	5a	Names, affiliations, and roles of protocol contributors	1
	5b	Name and contact information for the trial sponsor	1
	5c	Role of study sponsor and funders, if any, in study design; collection, management, analysis, and interpretation of data; writing of the report; and the decision to submit the report for publication, including whether they will have ultimate authority over any of these activities	N/A
	5d	Composition, roles, and responsibilities of the coordinating centre, steering committee, endpoint adjudication committee, data management team, and other individuals or groups overseeing the trial, if applicable (see Item 21a for data monitoring committee)	13 & 14
**Introduction**			

For peer review only - http://bmjopen.bmj.com/site/about/guidelines.xhtml

Background and rationale	6a	Description of research question and justification for undertaking the trial, including summary of relevant studies (published and unpublished) examining benefits and harms for each intervention	4-6
	6b	Explanation for choice of comparators	4-6
Objectives	7	Specific objectives or hypotheses	6
Trial design	8	Description of trial design including type of trial (eg, parallel group, crossover, factorial, single group), allocation ratio, and framework (eg, superiority, equivalence, noninferiority, exploratory)	7

Methods: Participants, interventions, and outcomes

Study setting	9	Description of study settings (eg, community clinic, academic hospital) and list of countries where data will be collected. Reference to where list of study sites can be obtained	7
Eligibility criteria	10	Inclusion and exclusion criteria for participants. If applicable, eligibility criteria for study centres and individuals who will perform the interventions (eg, surgeons, psychotherapists)	7
Interventions	11a	Interventions for each group with sufficient detail to allow replication, including how and when they will be administered	8
	11b	Criteria for discontinuing or modifying allocated interventions for a given trial participant (eg, drug dose change in response to harms, participant request, or improving/worsening disease)	8
	11c	Strategies to improve adherence to intervention protocols, and any procedures for monitoring adherence (eg, drug tablet return, laboratory tests)	10
	11d	Relevant concomitant care and interventions that are permitted or prohibited during the trial	N/A

For peer review only - http://bmjopen.bmj.com/site/about/guidelines.xhtml

Outcomes	12	Primary, secondary, and other outcomes, including the specific measurement variable (eg, systolic blood pressure), analysis metric (eg, change from baseline, final value, time to event), method of aggregation (eg, median, proportion), and time point for each outcome. Explanation of the clinical relevance of chosen efficacy and harm outcomes is strongly recommended	11
Participant timeline	13	Time schedule of enrolment, interventions (including any run-ins and washouts), assessments, and visits for participants. A schematic diagram is highly recommended (see Figure)	Figure is added
Sample size	14	Estimated number of participants needed to achieve study objectives and how it was determined, including clinical and statistical assumptions supporting any sample size calculations	10
Recruitment	15	Strategies for achieving adequate participant enrolment to reach target sample size	N/A
**Methods: Assignment of interventions (for controlled trials)**			
Allocation:			
Sequence generation	16a	Method of generating the allocation sequence (eg, computer-generated random numbers), and list of any factors for stratification. To reduce predictability of a random sequence, details of any planned restriction (eg, blocking) should be provided in a separate document that is unavailable to those who enrol participants or assign interventions	8
Allocation concealment mechanism	16b	Mechanism of implementing the allocation sequence (eg, central telephone; sequentially numbered, opaque, sealed envelopes), describing any steps to conceal the sequence until interventions are assigned	8

For peer review only - http://bmjopen.bmj.com/site/about/guidelines.xhtml

Implementation	16c	Who will generate the allocation sequence, who will enrol participants, and who will assign participants to interventions	8
Blinding (masking)	17a	Who will be blinded after assignment to interventions (eg, trial participants, care providers, outcome assessors, data analysts), and how	N/A, page 8
	17b	If blinded, circumstances under which unblinding is permissible, and procedure for revealing a participant's allocated intervention during the trial	N/A
**Methods: Data collection, management, and analysis**			
Data collection methods	18a	Plans for assessment and collection of outcome, baseline, and other trial data, including any related processes to promote data quality (eg, duplicate measurements, training of assessors) and a description of study instruments (eg, questionnaires, laboratory tests) along with their reliability and validity, if known. Reference to where data collection forms can be found, if not in the protocol	11
	18b	Plans to promote participant retention and complete follow-up, including list of any outcome data to be collected for participants who discontinue or deviate from intervention protocols	N/A
Data management	19	Plans for data entry, coding, security, and storage, including any related processes to promote data quality (eg, double data entry; range checks for data values). Reference to where details of data management procedures can be found, if not in the protocol	11
Statistical methods	20a	Statistical methods for analysing primary and secondary outcomes. Reference to where other details of the statistical analysis plan can be found, if not in the protocol	12
	20b	Methods for any additional analyses (eg, subgroup and adjusted analyses)	12
	20c	Definition of analysis population relating to protocol non-adherence (eg, as randomised analysis), and any statistical methods to handle missing data (eg, multiple imputation)	11

Methods: Monitoring

Data monitoring	21a	Composition of data monitoring committee (DMC); summary of its role and reporting structure; statement of whether it is independent from the sponsor and competing interests; and reference to where further details about its charter can be found, if not in the protocol. Alternatively, an explanation of why a DMC is not needed	12 & 16
	21b	Description of any interim analyses and stopping guidelines, including who will have access to these interim results and make the final decision to terminate the trial	11-12
Harms	22	Plans for collecting, assessing, reporting, and managing solicited and spontaneously reported adverse events and other unintended effects of trial interventions or trial conduct	16
Auditing	23	Frequency and procedures for auditing trial conduct, if any, and whether the process will be independent from investigators and the sponsor	N/A

Ethics and dissemination

Research ethics approval	24	Plans for seeking research ethics committee/institutional review board (REC/IRB) approval	17
Protocol amendments	25	Plans for communicating important protocol modifications (eg, changes to eligibility criteria, outcomes, analyses) to relevant parties (eg, investigators, REC/IRBs, trial participants, trial registries, journals, regulators)	N/A
Consent or assent	26a	Who will obtain informed consent or assent from potential trial participants or authorised surrogates, and how (see Item 32)	7-8
	26b	Additional consent provisions for collection and use of participant data and biological specimens in ancillary studies, if applicable	Mentioned in patient information form

For peer review only - http://bmjopen.bmj.com/site/about/guidelines.xhtml

Confidentiality	27	How personal information about potential and enrolled participants will be collected, shared, and maintained in order to protect confidentiality before, during, and after the trial	Mentioned in patient information form, page 14
Declaration of interests	28	Financial and other competing interests for principal investigators for the overall trial and each study site	20
Access to data	29	Statement of who will have access to the final trial dataset, and disclosure of contractual agreements that limit such access for investigators	A trial agreement has been made. Not mentioned in the manuscript.
Ancillary and post-trial care	30	Provisions, if any, for ancillary and post-trial care, and for compensation to those who suffer harm from trial participation	Mentioned in patient information form
Dissemination policy	31a	Plans for investigators and sponsor to communicate trial results to participants, healthcare professionals, the public, and other relevant groups (eg, via publication, reporting in results databases, or other data sharing arrangements), including any publication restrictions	16-17
	31b	Authorship eligibility guidelines and any intended use of professional writers	N/A
	31c	Plans, if any, for granting public access to the full protocol, participant-level dataset, and statistical code	N/A
**Appendices**			

For peer review only - http://bmjopen.bmj.com/site/about/guidelines.xhtml

Informed consent materials	32	Model consent form and other related documentation given to participants and authorised surrogates	Not attached, in dutch.
Biological specimens	33	Plans for collection, laboratory evaluation, and storage of biological specimens for genetic or molecular analysis in the current trial and for future use in ancillary studies, if applicable	12-15

For peer review only

2. Table 1: participating sites RAIN study

Amphia Hospital	Breda, The Netherlands
Erasmus MC-Sophia Children's Hospital	Rotterdam, The Netherlands
Groene Hart Hospital	Gouda, The Netherlands
Haaglanden Medical Center	Den Haag, The Netherlands
Haga/Juliana Children's Hospital	Den Haag, The Netherlands
Ikazia Hospital	Rotterdam, The Netherlands
Isala Klinieken	Zwolle, The Netherlands
Maasstad Hospital	Rotterdam, The Netherlands
Maxima Medical Center	Veldhoven, The Netherlands
Meander Medical Center	Amersfoort, The Netherlands
Medisch Spectrum	Twente, The Netherlands
Reinier de Graaf Hospital	Delft, The Netherlands
Rijnstate Hospital	Arnhem, The Netherlands
Sint Antonius Hospital *(2 locations)*	Nieuwegein & Utrecht, The Netherlands
Franciscus Gasthuis & Vlietland *(2 locations)*	Rotterdam & Schiedam, The Netherlands
IJsselland Hospital	Capelle aan den IJssel, The Netherlands

For peer review only

For peer review only - http://bmjopen.bmj.com/site/about/guidelines.xhtml

3. Risk assessment tables for suspected early-onset bacterial infection from the Dutch national guideline: *NVK Richtlijn: Preventie en behandeling van early-onset neonatale infecties*

Table 1: Risk factors for early-onset neonatal infection, including 'red flags

Risk factor	Red flag
**Parenteral antibiotic treatment given to the woman for confirmed or suspected invasive bacterial infection (such as septicaemia) at any time during labour, or in the 24-hour periods before and after the birth [This does not refer to intrapartum antibiotic prophylaxis]**	yes
**Suspected or confirmed infection in another baby in the case of a multiple pregnancy**	yes
Invasive group B streptococcal infection in a previous baby	
Maternal group B streptococcal colonisation, bacteriuria or infection in the current pregnancy	
Prelabour rupture of membranes >24 hours in term baby	
Preterm birth following spontaneous labour (before 37 weeks' gestation)	
Suspected or confirmed rupture of membranes for more than 18 hours in a preterm birth	
Intrapartum fever higher than 38°C, or confirmed or suspected chorioamnionitis	

Table 2 Clinical indicators of possible early-onset neonatal infection (observations and events in the baby), including 'red flags' Dutch national protocol. Adopted from the NICE guidelines 2012.

Clinical indicator	Red flag
**Respiratory distress starting more than 4 hours after birth**	yes
**Neonatal seizures**	yes
**Need for mechanical ventilation in a term baby**	yes
**Signs of shock**	yes
Altered behaviour or responsiveness (including floppiness)	
Feeding difficulties (including vomiting, excessive gastric aspirates and abdominal distension)	
Apnoea or bradycardia	
Signs of respiratory distress	
Hypoxia (for example, central cyanosis or reduced oxygen saturation level	
Signs of neonatal encephalopathy	
Need for cardio–pulmonary resuscitation	
Need for mechanical ventilation in a preterm baby	
Persistent fetal circulation (persistent pulmonary hypertension)	
Temperature abnormality (lower than 36°C or higher than 38°C) unexplained by environmental factors	
Signs of local infection (eyes, skin)	

References:

1. Neonatal infection (early onset): antibiotics for prevention and treatment [Internet]. 2012 [cited 28-08-2018]. Available from: https://www.nice.org.uk/guidance/cg149.

2. NVK Richtlijn: Preventie en behandeling van early-onset neonatale infecties, 2017. [cited 23-01-2019] Available from: https://www.nvk.nl/Kwaliteit/Richtlijnen-overzicht/Details/articleType/ArticleView/articleId/694/Preventie-en-behandeling-van-early-onset-neonatale-infecties#tab0

For peer review only - http://bmjopen.bmj.com/site/about/guidelines.xhtml

Survey 'ENGLISH: Survey day 7'

ENGLISH: Survey day 7 - Introduction

Dear parents/guardians, We have asked you permission to send two surveys as follow up on the RAIN study. This is the first survey, which has to be filled in around day 7 after initiation of therapy. Thank you for your help! RAIN team

ENGLISH: Survey day 7 - Survey day 7

Number	Question	Answers
1.1	Age mother	[ ] years
1.2	Age father	[ ] years
1.3	What is your highest degree in education?	○ I never finished school or training programme<br>○ Primary/Elementary school<br>○ Junior vocational eductaion<br>○ Lower genearal secondary education<br>○ Intermediate vocational education<br>○ Higher general secundary education<br>○ School for higher vocational education<br>○ University<br>○ Other degree
1.3.1	***If 'What is your highest degree in education?' is equal to 'Other degree' answer this question:***<br>If other, specify degree	[ ]
1.4	What is your highest degree in education?	○ I never finished school or training programme<br>○ Primary/Elementary school<br>○ Junior vocational eductaion<br>○ Lower genearal secondary education<br>○ Intermediate vocational education<br>○ Higher general secundary education<br>○ School for higher vocational education<br>○ University<br>○ Other degree
1.3.2	***If 'What is your highest degree in education?' is equal to 'Other degree' answer this question:***<br>If other, specify degree	[ ]

For peer review only - http://bmjopen.bmj.com/site/about/guidelines.xhtml

1.5	How many children (<18 years) do you have?	[ ] Children
1.6	Do they all live at home with you?	○ Yes<br>○ No
1.7	Do you, mother, have a paid job?	○ No<br>○ Yes, fulltime (>36 hours/week)<br>○ Yes, parttime
1.7.1	***If 'Do you, mother, have a paid job?' is equal to 'Yes, parttime' answer this question:***<br>If parttime, how many hours per week?	[ ] hours
1.7.2	***If 'Do you, mother, have a paid job?' is not equal to 'No' answer this question:***<br>Mother: What is (on average) your monthly salary (gross amount)?	○ Less than €999/month<br>○ Between €1.000 and 1.499/month<br>○ Between €1.500 and 1.999/month<br>○ Between €2.000 and 2.499/month<br>○ Between €2.500 and 2.999/month<br>○ Between €3.000 and 3.499/month<br>○ Between €3.500 and 3.999/month<br>○ Between €4.000 and 4.999/month<br>○ Between €5.000 and 5.999/month<br>○ Between €6.000 and 6.999/month<br>○ Between €7.000 and 7.999/month<br>○ Between €8.000 and 8.999/month<br>○ Between €9.000 and 9.999/month<br>○ More than €10.000/month<br>○ I don't know, I do not want to tell this
1.8	Do you, mother, have an unpaid job (except for caregiving)?	○ Yes<br>○ No
1.8.1	***If 'Do you, mother, have an unpaid job (except for caregiving)?' is equal to 'Yes' answer this question:***<br>if yes, for how many hours per month?	[ ]
1.9	Do you, father, have a paid job?	○ No<br>○ Yes, fulltime (>36 hours/week)<br>○ Yes, parttime
1.9.1	***If 'Do you, father, have a paid job?' is equal to 'Yes, parttime' answer this question:***<br>If parttime, how many hours per week?	[ ] hours

For peer review only - http://bmjopen.bmj.com/site/about/guidelines.xhtml

1.9.2	***If 'Do you, father, have a paid job?' is not equal to 'No' answer this question:***<br>Father: What is (on average) your monthly salary (gross amount)?	Less than €999/month<br>Between €1.000 and 1.499/month<br>Between €1.500 and 1.999/month<br>Between €2.000 and 2.499/month<br>Between €2.500 and 2.999/month<br>Between €3.000 and 3.499/month<br>Between €3.500 and 3.999/month<br>Between €4.000 and 4.999/month<br>Between €5.000 and 5.999/month<br>Between €6.000 and 6.999/month<br>Between €7.000 and 7.999/month<br>Between €8.000 and 8.999/month<br>Between €9.000 and 9.999/month<br>More than €10.000/month<br>I don't know, I do not want to tell this
1.10	Do you, father, have an unpaid job (except for caregiving)?	Yes<br>No
1.10.1	***If 'Do you, father, have an unpaid job (except for caregiving)?' is equal to 'Yes' answer this question:***<br>Father: if yes, for how many hours per month?	
1.11	Did you hire extra baby-sitting because of the hospital admission of your child?	Yes<br>No
1.12	Did you partner, in the past week, work less than expected in order for him tot take care of you and/or other children because of the admission?	Yes<br>No
1.12.1	***If 'Did you partner, in the past week, work less than expected in order for him tot take care of you and/or other children because of the admission?' is equal to 'Yes' answer this question:***<br>If yes, how many hours less than expected?	

1.13	For how many days was your baby admitted to the hospital?	○ 2.5 days<br>○ 3 days<br>○ 3.5 days<br>○ 4 days<br>○ 4.5 days<br>○ 5 days<br>○ 5.5 days<br>○ 6 days<br>○ 6.5 days<br>○ 7 days<br>○ 7.5 days<br>○ 8 days<br>○ > 8 days
1.14	How many times did your baby receive a new IV canula?	○ Not, because he/she was treated with antibiotic suspension<br>○ 1 x<br>○ 2 x<br>○ 3 x<br>○ 4 x<br>○ More often<br>○ I do not remember<br>○ My child only kept the first inserted canula for the whole treatment
1.15	How high were your travel expenses each day for travelling from your house to the hospital?	[ ] euro
1.16	Did you visit another doctor this week for your baby?	○ Yes<br>○ No
1.16.1	***If 'Did you visit another doctor this week for your baby?' is equal to 'Yes' answer this question:***<br>If yes, what kind of doctor?	[ ]
1.17	Did your baby experience fever this week?	○ Yes<br>○ No
1.17.1	***If 'Did your baby experience fever this week?' is equal to 'Yes' answer this question:***<br>If yes, how high was the fever?	[ ] degrees Celcius

For peer review only - http://bmjopen.bmj.com/site/about/guidelines.xhtml

1.18	Did your child experience any side effects of the antibiotic therapy?	○ Vomiting<br>○ Change is defecation pattern<br>○ Rash<br>○ Other<br>○ None
1.19	What kind of feeding do you give to your baby?	○ Breastmilk<br>○ Formula milk<br>○ Combination of breast- and formulamilk
1.20	How does your child sleep?	○ Goes to sleep easily and wakes up satisfied<br>○ Going to sleep is sometimes difficult, but does eventually sleep well<br>○ Going to sleep is difficult, cries often, hard to comfort
1.21	Did your baby receive any other medication (besides vitamins)?	○ Yes<br>○ No
1.21.1	***If 'Did your baby receive any other medication (besides vitamins)?' is equal to 'Yes' answer this question:***<br>If yes, which medication?	

ENGLISH: Survey day 7 - Outro

Thank you for helping us by filling in this survey. You will receive the second survey in two weeks. Kind regards, RAIN team

Survey 'ENGLISH survey day 21'

ENGLISH survey day 21 - Introduction

Dear parents/guardians, We have asked you permission to send two surveys as follow up on the RAIN study. This is the second survey, which has to be filled in around 3 weeks after initiation of therapy. Thank you for your help! RAIN team

ENGLISH survey day 21 - ENGLISH survey day 21

Number	Question	Answers
1.1	Did you visit another doctor this week for your baby?	○ Yes<br>○ No
1.1.1	***If 'Did you visit another doctor this week for your baby?' is equal to 'Yes' answer this question:***<br>If yes, what kind of doctor?	
1.2	Did your baby experience fever this week?	○ Yes<br>○ No
1.2.1	***If 'Did your baby experience fever this week?' is equal to 'Yes' answer this question:***<br>If yes, how high was the fever?	degrees Celcius
1.3	What kind of feeding do you give to your baby?	○ Breastmilk<br>○ Formula milk<br>○ Combination of breast- and formulamilk
1.4	How does your child sleep?	○ Goes to sleep easily and wakes up satisfied<br>○ Going to sleep is sometimes difficult, but does eventually sleep well<br>○ Going to sleep is difficult, cries often, hard to comfort
1.5	Did your baby receive any other medication (besides vitamins)?	○ Yes<br>○ No
1.5.1	***If 'Did your baby receive any other medication (besides vitamins)?' is equal to 'Yes' answer this question:***<br>If yes, which medication?	

For peer review only - http://bmjopen.bmj.com/site/about/guidelines.xhtml

ENGLISH survey day 21 - Outro

Thank you for helping us by filling in this survey. This was the last survey of the RAIN study. Kind regards, RAIN team

For peer review only - http://bmjopen.bmj.com/site/about/guidelines.xhtml

Survey 'ENGLISH: microbiome T = 6 months'

ENGLISH: microbiome T = 6 months - Introduction

Dear parents/guardians, You have agreed to collect fecal samples in the first year of life of your child. It is time to collect a sample for the 4th time. 1. Please collect some stool from the diaper of your baby in the tube with the label: RAIN FECES 4 T = 6 months and put it directly back into the freezer. 2. Please answer the following questions.

ENGLISH: microbiome T = 6 months - questionnaire microbiome T = 6 months

Number	Question	Answers
1.1	What kind of feeding do you give to your child?	Breastmilk<br>Formula milk<br>Combination of breast- and formulamilk
1.2	Did you child start with solid food yet?	Yes<br>No
1.3	Are there older children living with you and your child in the same house?	no<br>yes, 1<br>yes, 2<br>yes, 3<br>yes, more than 3
1.3.1	***If 'Are there older children living with you and your child in the same house?' is not equal to 'no' answer this question:***<br>If yes, what age group are they in?	< 4 years old<br>4-12 years old<br>>12 years old
1.3.1.1	***If 'If yes, what age group are they in?' is equal to '< 4 years old' answer this question:***<br>Are your children, younger than 4 years old, going to daycare?	No<br>Yes, 1 day /week<br>Yes, 2 days /week<br>Yes, more than 2 days/week

1.4	Did your child experience any episodes of illness in the last 3 months?	☐ No<br>☐ Yes, nasal congestion/stuffy nose<br>☐ Yes, coughing<br>☐ Yes, fever > 38.0 degrees celcius<br>☐ Yes, diarrhea<br>☐ Yes, skin rash for more than 1 day
1.4.1	***If 'Did your child experience any episodes of illness in the last 3 months?' is not equal to 'No' answer this question:***<br>If yes, did you visit a doctor?	○ Yes<br>○ No
1.4.1.1	***If 'If yes, did you visit a doctor?' is equal to 'Yes' answer this question:***<br>If yes, please specify who (example: general practitioner, pediatrician)	
1.5	Has your child been readmitted to the hospital in the last 3 months?	○ Yes<br>○ No
1.5.1	***If 'Has your child been readmitted to the hospital in the last 3 months?' is equal to 'Yes' answer this question:***<br>If yes, for what reason?	
1.6	Did your child receive another course of antibiotics in the past 3 months?	○ Yes<br>○ No
1.6.1	***If 'Did your child receive another course of antibiotics in the past 3 months?' is equal to 'Yes' answer this question:***<br>If you remember the name of the treatment, please write it down	

ENGLISH: microbiome T = 6 months - Outro

In 6 months you will receive the last questionnaire and collect the last stool sample. Thank you for your help! RAIN team

BMJ Open

RAIN study: a protocol for a randomized controlled trial evaluating efficacy, safety and cost-effectiveness of intravenous-to-oral antibiotic switch therapy in neonates with a probable bacterial infection

Journal:	*BMJ Open*
Manuscript ID	bmjopen-2018-026688.R2
Article Type:	Protocol
Date Submitted by the Author:	02-Apr-2019

Complete List of Authors:	Keij, Fleur; Erasmus MC Sophia, Pediatrics, Division of Neonatology<br>Kornelisse, René; Erasmus MC Sophia, Pediatrics, Division of Neonatology<br>Hartwig, Nico; Sint Franciscus Vlietland Groep, Pediatrics<br>Mauff, Katya; Erasmus MC, Biostatistics<br>Poley, Marten; Erasmus MC Sophia, Pediatric Surgery; Erasmus Universiteit Rotterdam, Medical Technology Assessment (iMTA)<br>Allegaert, Karel; Erasmus MC Sophia, Pediatrics, Division of Neonatology<br>Reiss, Irwin; Erasmus MC Sophia, Pediatrics, Division of Neonatology<br>Tramper-Stranders, Gerdien; Erasmus MC Sophia, Pediatrics, Division of Neonatology; Sint Franciscus Vlietland Groep, Pediatrics

**Primary Subject Heading**:	Paediatrics
Secondary Subject Heading:	Infectious diseases
Keywords:	amoxicillin-clavulanic acid, intravenous-to-oral antibiotic switch therapy, cost-effectiveness, microbiome, neonatal infections

SCHOLARONE™ Manuscripts

RAIN study: a protocol for a randomized controlled trial evaluating efficacy, safety and cost-effectiveness of intravenous-to-oral antibiotic switch therapy in neonates with a probable bacterial infection

Fleur M. Keij (MD)[1,2], René F. Kornelisse (PhD)[1], Nico G. Hartwig (PhD)[2], K. Mauff (Msc)[3] , Marten J. Poley (PhD)[4,5], Karel Allegaert (PhD)[1], Irwin K. M. Reiss (PhD)[1], Gerdien A. Tramper-Stranders (PhD)[1,2]

[1]Department of Pediatrics, Division of Neonatology, Erasmus MC- Sophia Children's Hospital, Rotterdam, The Netherlands
[2]Department of Pediatrics, Franciscus Gasthuis & Vlietland, Rotterdam, The Netherlands
[3]Department of Biostatistics, Erasmus MC - University Medical Centre, Rotterdam, The Netherlands
[4] Intensive care and Department of Pediatric Surgery, Erasmus MC - Sophia Children's Hospital, Rotterdam, The Netherlands
[5] Institute for Medical Technology Assessment (iMTA), Erasmus University Rotterdam, Rotterdam, The Netherlands

Correspondence to:

Fleur M. Keij (MD)

Department of Pediatrics, Division of Neonatology, Erasmus MC- Sophia Children Hospital

3015 CN Rotterdam, The Netherlands

f.keij@erasmusmc.nl

For peer review only

For peer review only - http://bmjopen.bmj.com/site/about/guidelines.xhtml

1. Abstract

Introduction: High morbidity and mortality rates of proven bacterial infection are the main reason for substantial use of intravenous antibiotics in neonates during the first week of life. In older children, intravenous-to oral switch after 48 hours of intravenous therapy has been shown to have many advantages and is nowadays commonly practiced. We therefore aim to evaluate the effectiveness, safety, and cost-effectiveness of an early intravenous to oral switch in neonates with a probable bacterial infection.

Methods and analysis: We present a protocol for a multicenter randomised trial assessing the non-inferiority of an early intravenous- to oral antibiotic switch compared to a full course of intravenous antibiotics in neonates (0-28 days of age) with a probable bacterial infection.

Five hundred-and fifty patients will be recruited in 17 hospitals in the Netherlands. After 48 hours of intravenous treatment, they will be assigned to either continue for another 5 days with intravenous therapy (control) or switch to amoxicillin/clavulanic acid suspension for 5 days (intervention). Both groups will be treated for a total of 7 days. The primary outcome will be bacterial (re-)infection within 28 days after treatment completion. Secondary outcomes are the pharmacokinetic profile of oral amoxicillin/clavulanic acid, the impact on quality of life, cost-effectiveness, impact on microbiome development and additional yield of molecular techniques in diagnosis of probable bacterial infection.

Ethics and dissemination: This study has been approved by the Medical Ethics Committee of the Erasmus Medical Center. Results will be presented in peer-reviewed journals and at international conferences.

Registration: The trial has been registered in Clinicaltrials.gov Trial number NCT03247920.

For peer review only - http://bmjopen.bmj.com/site/about/guidelines.xhtml

2. Strengths and limitations of the study

Max five short bullet points, no longer than one sentence each, that relate specifically **to the methods.**

- This study addresses an important clinical question regarding intravenous-to-oral antibiotic switch in neonatal care
- The results of this study can be included in antibiotic stewardship programs
- A non-inferiority study design is used
- The primary outcome assessment is challenging since it is impossible to state whether a new infection episode is a true re-infection since cultures of the first infection were sterile.
- Only probable bacterial infections are included in this study, thus results can not directly be applied to culture-proven infection.

For peer review only

For peer review only - http://bmjopen.bmj.com/site/about/guidelines.xhtml

3. Introduction

Antibiotics are among the most prescribed drugs in the pediatric population. With increasing concerns regarding microbial resistance leading to potential multi-drug resistant infections, antibiotic stewardship programs (ASPs) have been developed. The main goal of these programs is optimization of the use of antibiotics. Moreover, unnecessary use should be reduced since antibiotics do interfere with the development of the gut microbiome in early stages of life, influencing the risk of developing several diseases such as asthma, diabetes and obesity later in life (1-3). Initially started in adult population, these programs have, since then, been implemented in the pediatric field, starting with the formation of the Pediatric Committee on Antimicrobial Stewardship (AS) by the Pediatric Infectious Diseases Society (PIDS) in 2010. One of the principles of AS is the appropriate and timely de-escalation of antibiotic use including a switch from parenteral to oral therapy in clinically well children. An early intravenous-to-oral switch leads to many advantages such as a reduction in hospital stay and associated health costs and is currently described in several guidelines. (4)

However, oral switch therapy is not yet widely practiced in neonates. When a bacterial infection is suspected, either based on maternal risk factors or subtle clinical symptoms, antibiotic therapy is immediately started. In the best-case scenario, symptoms disappear, inflammatory parameters are reassuring and cultures remain negative after which therapy can be stopped after 36-48 hours. In some of the neonates (~1% of living births) the presence or absence of a bacterial infection cannot be established. (5) They may have elevated inflammatory parameters and show clinical signs, although (blood) cultures remain sterile; so-called “probable bacterial infection”. In such cases, antibiotic therapy is usually continued intravenously, up to seven days. (6)

For peer review only - http://bmjopen.bmj.com/site/about/guidelines.xhtml

In Low and Middle Income countries, the above practice is not common due to limited resources. In order to reduce mortality following neonatal infections there is a need for good home-based antibiotic treatment which has led to several large randomized trials on the use of oral antibiotics. These studies showed that a simplified regimen with a switch to oral amoxicillin after 48 hours is as effective and safe as parenteral therapy. (7-9) Results of those studies have been incorporated in the WHO guidelines for the management of neonatal sepsis. (10) Unfortunately, the outcomes cannot be applied to a higher income setting since the studies were based on clinical symptoms solely and no diagnostic tests were performed. This approach has not yet led to further research in High Income Settings.

Several small studies have been published on the use of different oral antibiotics, evaluating both pharmacokinetics and clinical efficacy. Regarding pharmacokinetics, studies have commonly found that adequate antibiotic serum levels can be reached following oral administration, although peak-levels are lower and appear later in time compared to parenteral administration. Furthermore a great inter individual variation was seen. (11, 12) Small efficacy studies evaluating an early oral switch in neonates with a possible or proven infection are promising. In a cohort study, 222 infants with possible and proven group B streptococcal (GBS) infection switched to oral amoxicillin in a high dosage after 48 hours of IV treatment. During the three-month follow up period, no reinfections occurred. (13) Similar results were found in a case-control study in which 36 infants with a possible or proven bacterial infection switched to oral cefpodoxime on day 3. No increase in reinfection rate was observed and the benefits of oral therapy included reduction of hospital stay and an increase in the number of exclusively breastfed newborns. (13, 14) Unfortunately, study sizes were small and studies were not randomized. Furthermore, no full cost-effectiveness analyses of pediatric intravenous-to-oral switch therapy have been reported to date. This represents an important knowledge gap, considering that, in the current era

For peer review only - http://bmjopen.bmj.com/site/about/guidelines.xhtml

of austerity and rising health care costs, it is increasingly required to demonstrate whether or not new treatment strategies offer good value for money.

Together with uncertainties about bioavailability after oral administration, the lack of firm evidence delays further implementation in a high resource setting. When intravenous-to-oral antibiotic switch therapy in neonatal infections is proven safe and effective, it can lead to a great improvement in quality of life for both child and parents. Furthermore, it can reduce the length of hospital stay, which will have positive effects on rising health care costs.

Objectives

The primary objective is to demonstrate non-inferiority of intravenous-to-oral antibiotic switch therapy in clinically well neonates with a probable bacterial infection compared to a complete course of intravenous therapy. The secondary objectives are: 1) to describe pharmacokinetics of oral amoxicillin clavulanic acid in neonates; 2) to quantify the cost-effectiveness of intravenous-to-oral switch therapy in neonates; 3) to evaluate the quality of life in relation to switch therapy in neonates; 4) to study modification of the gut microbiome in relation to type and administration of antibiotics and 5) to explore the additional value of molecular diagnostics on bacterial infection using left over blood culture material that remains after culture-time has passed.

For peer review only - http://bmjopen.bmj.com/site/about/guidelines.xhtml

4. Methods and Analysis

Study design

The RAIN (**R**eduction of intravenous **A**ntibiotics **I**n **N**eonates) study is a multicenter, randomized controlled, open label, non-inferiority trial. Patients will be recruited in 17 large teaching hospitals in the Netherlands. For details on participating hospitals we refer to the supplementary material.

For peer review only

Participants

Neonates are eligible for participation if they have a postmenstrual age of 35 weeks or more, are 0-28 days old, have a body weight of at least two kilograms and have a “probable bacterial infection” based on clinical symptoms or maternal risk factors and elevated inflammatory parameters for which the pediatrician has decided to complete a full course of intravenous antibiotics. A C-reactive protein level of ≥ 10 mg/L is considered as elevated. For procalcitonin (PCT), we refer to the age-adapted ranges. (15, 16) Clinical symptoms can be found in the supplementary material. All neonates with a proven bacterial infection, e. g. positive blood- or cerebrospinal fluid culture or a severe clinical sepsis requiring intensive care treatment are excluded from participation. In- and exclusion criteria are described in table 1. Participants will be screened by the local pediatrician or investigator and randomized after 48 hours of intravenous treatment after informed consent has been obtained.

Inclusion Criteria

- Neonates, postmentrual age ≥ 35+0 weeks, postnatal age of 0-28 days, body weight ≥ 2 kg
- Probable bacterial infection defined as clinical symptoms and/or maternal risk factors *and* elevated inflammatory markers for which empiric broad-spectrum antibiotic treatment was initiated and needs to be continued for > 48 hours
- Clinically well
- Tolerate oral feeding without overt vomiting

Exclusion Criteria

- Proven bloodstream infection
- Absence of blood culture
- Severe localized infection (meningitis, osteomyelitis, necrotizing enterocolitis)
- Severe clinical sepsis (compromised circulation, need for mechanical ventilation)
- Continuous need for a central venous line
- Severe hyperbilirubinemia exceeding the exchange level
- Parents’ inability to administer medication

Table 1: Inclusion and exclusion criteria RAIN study

Intervention

The intervention group will switch to oral amoxicillin/clavulanic acid after between 48-72 hours of intravenous antibiotics. The number of intravenous antibiotic doses prior to the switch will be recorded for every participant. The control group will receive the full course of intravenous antibiotics, which consists of penicillin and gentamicin in case of early onset sepsis in most participating centers. Both groups will be treated for a total of 7 days. The trial flow diagram is outlined in figure 1.

Dosage, method of administration

A daily oral dose of 75/18.75 mg/kg of amoxicillin/clavulanic acid, thus 25/6.25 mg/kg every 8 hours, will be administered. The mode of administration is orally via suspension, or in case of feeding problems (with the exception of vomiting) per nasopharyngal tube. If the neonate vomits significantly within 30 minutes after administration, the administration will be repeated.

Randomization and blinding

Participants will be randomized after 48 hours of intravenous treatment by a web-based program according to a stratified block randomization (stratified per site in alternated blocks of 2 and 4). Randomization ratio will be 1:1. Randomization can under no circumstances be influenced by the study team. If parents want their child to be in a specific group they will be excluded from participation. Patients and health care professionals will not be blinded, because of the nature of treatments (intravenous versus oral therapy); the primary investigators performing the statistical analysis will be blinded for the allocated treatment.

Follow up

For peer review only - http://bmjopen.bmj.com/site/about/guidelines.xhtml

Neonates will only be discharged from the hospital if they have shown to tolerate the oral antibiotic treatment for at least 2 doses and are clinically well. Parents will be interviewed by telephone on the first day after discharge and on day 14 after initiation of antibiotic therapy by the local study investigator or research nurse. The local investigator will assess antibiotic intake, signs of infection, side effects and events. In addition, parents will receive a survey on day 7 and day 21 with questions regarding possible signs of infection, side effects, quality of life, loss of productivity, and cost effectiveness and demographic data (maternal, paternal age, highest level of education, socio-economic status and siblings). All surveys are available as supplementary material. The primary outcome of the study is assessed 28 days after treatment completion, when the patient will be seen by the local investigator or research nurse for a final visit at the outpatient department. Parents of participants in the oral treatment group will be asked to return the bottle with remaining antibiotic suspension in order to evaluate medication adherence through measurement of the remaining amount of medication present in the bottle. Follow up of neonates in the control group will be identical.

Power calculation

Relapse of proven Group B streptococcus (GBS) infection is estimated to occur in 0-8% of patients, but may be caused by a different strain. (17) Two epidemiological studies estimated the recurrence rate to be 1% in proven bacterial neonatal infections. (18, 19) The current study focuses on probable infections (in which the recurrence rate is likely even lower, although evidence for this is lacking). The incidence of community-acquired late onset sepsis in otherwise healthy neonates is low (~0.28 per 1000 livebirths).(20) Therefore, we assume a 1 % infection rate (including new non-related infections) in both groups. Based on a non-inferiority margin of 3% (minimum success rate 96 %), 2 x 231 patients are needed for a power of 90% using a 1-sided alpha of 0.025. Because of the first 30 patients undergoing PK measurements and uncertainty about the PK values, and potential dropout or withdrawal (estimated

For peer review only - http://bmjopen.bmj.com/site/about/guidelines.xhtml

approximately 5-10%), we will include 2 x 275 neonates (total = 550). A post-hoc report on the design effect will be included.

Data collection and management

Data will be collected by the local investigator at each site and stored anonymously in a GCP proof digital database (Castor EDC, Cewit B.V). Two independent monitors will perform source data verification and assess performance of study procedures at each site following a predefined schedule.

Outcome measures and statistical analysis

Primary outcome

The primary outcome is bacterial (re-) infection within 28 days after finishing antimicrobial therapy, defined as clinical symptoms of infection and fever (> 38.0 °C) or hypothermia (< 36.0 °C) and elevated inflammatory parameters with a need for prolonged antimicrobial therapy for more than 48-72 hours. Proven or suspected viral infection, for which no antibiotics are initiated or antibiotics are stopped within 48-72 hours (after return of culture and/or viral results) are not considered as a bacterial (re-) infection. A chart will aid in decision making with respect to differentiation between bacterial infection and other causes. Both per-protocol analysis and intention-to-treat analysis will be performed. A p-value <0.025 will be considered as significant. Regarding the primary outcome, 28-day cumulative bacterial infection rate; proportions will be defined and used to compare both treatment strategies. Subgroup analysis will be performed on late preterm neonates (GA 35-37 weeks). Further, mixed effect models will be used to take into account the multiple centers.

Interim analysis

For peer review only - http://bmjopen.bmj.com/site/about/guidelines.xhtml

A formal interim analysis will be performed after occurrence of the first four primary outcomes. The Data Safety Monitoring Board (DSMB) will assess the results and it will be determined whether the occurrence of the primary outcome was related to the index suspected bacterial infection or more likely to be an unrelated infection. The outcome of that interim analysis will determine after how many primary outcomes the next interim analysis will be performed. When more than 75% of related cases occur in the intervention arm in two consecutive interim analyses, the study will be stopped prematurely.

Secondary outcomes and their statistical analyses are described below.

<u>Secundary outcomes</u>

Pharmacokinetics

Blood samples of the first 30 patients treated with oral antibiotics (intervention arm) will be analyzed in order to confirm that plasma concentrations are within target range and to endorse safety and dosing of the investigated product. It is thought that time above MIC should be at least 40-50% in order for treatment with penicillins to be adequate. (21) Therefore, T>MIC should be at least 50% in 90% of the cases to be considered adequate, otherwise dosing needs to be adjusted. A target MIC of 8 mg/l is chosen for amoxicillin since this is the clinical breakpoint for *E. coli* susceptibility defined by the European Committee on Antimicrobial Susceptibility (Eucast). (22) In a later stage, sampling will be continued in order to develop a PK/PD model and to perform a population pharmacokinetic analysis. For the first 30 patients, two blood samples (0.5 ml whole blood) will be collected; the first sample 4-6 hours after the first or second dose, the second sample 6-8 hours after the second or third dose of oral suspension. In a subset of patients, we will collect 2 samples following the same administration in order to calculate an area under the curve (AUC). After the first analysis on samples from 30 patients;

For peer review only - http://bmjopen.bmj.com/site/about/guidelines.xhtml

sampling will be continued through the collection of a single blood sample per patient. Samples will be handled within one hour after collection by the local laboratory. The sample will be centrifuged at 3000 rpm for ten minutes and the recovered plasma will be stored at -80°C. Once enough samples have been collected for analysis, they will be shipped to the Erasmus MC for analysis. Serum concentrations of both amoxicillin and clavulanic acid will be measured. For each measurement 50 microliters of plasma is needed. Analysis will be performed batchwise combining both Liquid Chromatography (LC) and Mass Spectometry (MS). Analysis will be performed by descriptive statistics and by using nonlinear mixed effects modeling (NONMEM) including variables such as timing of blood drawing, timing of last meal, patient weight, center.

Molecular techniques for bacterial detection

Left over material of blood culture before initiation of antibiotic treatment will be stored at -80°C. Molecular analysis will be performed using next generation sequencing (NGS) methods, evaluating the additional yield of those techniques on bacterial detection compared to blood culture. Analysis will be performed batchwise, meaning that the additional value will be determined afterwards and that results will not be immediately available for the treating clinician nor for the DSMB in case of a (re-)infection. However results will be available at the end of the study and will be analysed in regard to primary outcomes that have occurred during the course of the study. The analysis of the Next Gen Sequencing (NGS) will initially be exploratory. If substantial differences are noted in the yield of recovered organisms from NGS compared to the blood culture, further analysis may be undertaken, exploring the relationship with clinical data such as inflammatory parameters and clinical illness. In that instance, mixed models will be used, and adjustments will be made for center.

Gut microbiome

For peer review only - http://bmjopen.bmj.com/site/about/guidelines.xhtml

Microbiome alterations following antibiotic therapy, both in the intervention and control group, will be evaluated in 80 participants from four centers. Fecal samples will be collected at 5 different moments in the first year of life (around day 7, 30, 90, 180 and day 365) from neonates born vaginally. Samples will be collected from the diaper and immediately stored at -20 °C in a special box placed in the home freezer. Parents will receive a notification at each sampling moment including a questionnaire regarding episodes of antibiotic treatment, type of feeding, daycare attendance and the presence of siblings at home. After 365 days, all samples will shipped to the central research locations and stored at -80°C until analysis in a specialized facility using high throughput sequencing targeted at the bacterial 16S rRNA locus. Alpha diversity of all samples will be calculated by the Shannon index. We will identify time points in which the alpha diversity and relative abundances in operational taxonomical units differ between the infants with iv-treatment and infants with iv-oral switch with a smoothing spline analysis of variance (SS-ANOVA). Moreover, differences in the relative abundances of the genera will be tested with a linear mixed effect model with correction for the variables of other antibiotic use, feeding type, day care attendance/sibling and center.

Cost-effectiveness analysis

Following a superiority design, the cost-effectiveness analysis (CEA) will make a comparison between the intervention and the control group by identifying, measuring and valuing the costs and patient outcomes of both treatment strategies.

Following established methods for economic evaluations and costing studies in health care, both medical costs and non-medical costs (i.e., costs of productivity losses) will be analyzed for both study groups. (23, 24) Total medical costs over the 28-day follow-up period will be calculated, including costs of intravenous antibiotics, costs of oral antibiotics, laboratory costs and costs of hospitalization days. In

For peer review only - http://bmjopen.bmj.com/site/about/guidelines.xhtml

regard to readmission costs, only costs of a readmission for a bacterial (re-) infection, as defined in the outcome measures section, will be added to the analysis. Resource consumption will be derived from electronic hospital databases. Unit prices will be calculated using real economic cost prices or using standard cost prices for health economic evaluations. Unit prices will be multiplied by the quantities for each resource used, and then summed over the separate types of resource to give a total cost per patient. Then, productivity costs in the patients' parents/caregivers, consisting of productivity losses regarding both paid and unpaid work, will be analyzed (ignoring prearranged, regular maternity and paternity leave). These costs will be measured using the institute of Medical Technology Assessment (iMTA) Productivity Cost Questionnaire (iPCQ) (25), completed by the parents during the scheduled follow up as described above. The productivity costs will be valued following the friction cost method. Briefly, the valuation of paid work will be based on average hourly labor costs per paid employee, whereas that of unpaid work will be based on the replacement costs for household work. Finally, all costs will be summated for each individual patient.

Regarding the patient outcomes, the CEA will look at the number of bacterial (re-) infections within 28 days after finishing of antimicrobial therapy. Building on the data on costs and outcomes described above, a cost-effectiveness ratio will be calculated (the final end-point of the cost-effectiveness analysis). This ratio will be expressed as incremental costs per bacterial (re-)infection avoided. Otherwise, the CEA will focus on dominance of one treatment over the other with respect to lower cost and greater effect. Analysis of uncertainty in the estimation of the incremental cost-effectiveness ratio is illustrated through a cost-effectiveness plane (via bootstrapping). Where relevant, sensitivity analysis will be performed to assess the robustness of the analysis to certain assumptions.

Quality of life

For peer review only - http://bmjopen.bmj.com/site/about/guidelines.xhtml

To get an impression of the impact of an early intravenous-to-oral antibiotic switch on the quality of life (QoL) of both parents and their child, parents will be asked to fill in a questionnaire on day 7 and day 21. Since there is no validated QoL instrument for neonates < 1 month, we developed a questionnaire ourselves that collects information on total admission days and readmission rate, symptoms, side effects, amount of intravenous cannulas inserted during admission, sleep quality of the patient, number of medical visits and medication use in the first month, feeding and breastfeeding success rate and parents' satisfaction. Outcome reporting will be mainly descriptive, if a trend is observed for some of the questions, mixed effect models can be used to further explore the effect of treatment and center.

Safety consideration

Serious life events (SAE) will be reported to the principal investigator within 48 hours. The principal investigator will report the adverse events to the Medical Ethics Committee (METC) of the Erasmus Medical Center and the Central Committee on Research Involving Human Subjects (CCMO). All SAEs will be followed until they have abated, or until a stable situation has been reached. An independent DSMB will be installed consisting of an epidemiologist/statistician, consultant neonatologist and paediatric infectious diseases specialist. The DSMB will evaluate the progress of the trial and will examine safety parameters at yearly intervals and through the performance of interim analyses when necessary. The adverse events will be listed and discussed with the DSMB.

Patient and Public Involvement

The Dutch parent organisation "Vereniging van Ouders van Couveusekinderen (VOC)" was involved in the conceptualization of the trial and has given their approval for the execution of this study. Patients will be asked at inclusion if they want to be informed on the final study results by email. Furthermore, a summary of findings will be published on the website of the VOC and on our own website.

For peer review only - http://bmjopen.bmj.com/site/about/guidelines.xhtml

For peer review only

5. Ethics and dissemination

This study was approved by the Medical Ethics Committee of the Erasmus Medical Center (NL51888.078.16, June 2017). The study will be conducted according to the principles of the Declaration of Helsinki (7th revision, October 2013) and in accordance with the Medical Research Involving Human Subjects Act (WMO) and other guidelines, regulations and Acts such as Good Clinical Practice.

The trial has been registered in Clinicaltrials.gov Trial number NCT03247920. The results of the primary and secondary aims will be published in peer-reviewed journals and presented at international conferences, followed by a press release.

For peer review only

For peer review only - http://bmjopen.bmj.com/site/about/guidelines.xhtml

6. Discussion/Conclusion

Prolonged hospitalization for intravenous therapy is still common practice in high income societies when neonatal bacterial infection cannot be ruled out despite negative cultures. In-hospital treatment has a high impact on both patient and parents since prolonged hospitalisation often leads to infant-parent separation. Initiatives to prevent separation through the reduction of admission days are of increasing interest. The additional value of other biomarkers besides CRP, such as procalcitonin, in order to rule out bacterial infection has been evaluated. A recent study showed that using procalcitonin values as guidance for antibiotic therapy in neonates with a suspected bacterial infection leads to a reduction in days on therapy and hospitalization. However, when the infection could not be ruled out, as is the case in infants with a probable infection, therapy was still continued intravenously for seven days. (26) We propose the first randomized controlled trial in a high resource setting with a non-inferiority study design in order to demonstrate that oral antibiotic switch therapy is as effective as intravenous treatment in neonates with a probable bacterial infection. Furthermore, we will be the first to perform PK/PD analysis on oral amoxicillin/clavulanic acid on a large sample size. Once proven that serum concentrations following oral administration are adequate, and non-inferiority of oral switch has been proven, oral switch therapy could potentially be expanded to clinically well infants with a proven infection.

This study has some limitations, the most important limitation relating to the primary outcome assessment. As mentioned in the methods section, only neonates with a probable bacterial infection will be included. It is therefore impossible to state whether or not a second infection is a reinfection or that it is caused by a different pathogen. We therefore developed a list of recommended diagnostics that should be determined when a participant returns with signs of a possible infection. Those results can be

For peer review only - http://bmjopen.bmj.com/site/about/guidelines.xhtml

used for correct and uniform assessment of the event and will help differentiating between a true bacterial infection, a probable bacterial infection or a viral infection.

Another limitation of the study relates to the fact that pharmacokinetic blood sampling will be performed in the intervention group. As this procedure carries a small risk of infections, bias may be introduced into the study. On the other hand, patients in the control group are at risk for infection when replacement or new insertion of an intravenous cannula is needed. Both situations can lead to a possible bacterial infection. We think, however, that the infection risk is low when performed correctly including good hygiene measurements. Moreover, neonates in both groups are under antibiotic treatment which also covers *S. aureus*.

Finally, bias may occur because of the oral treatment being administered out of the hospital setting. Since this treatment is home-based, parents' compliance in administering medication may be suboptimal, and we might underestimate the effectiveness of the intervention. In an attempt to overcome this limitation, infants will only be discharged when parents have shown to adequately administer the antibiotic suspension, parents will be phoned 1-2 days after discharge to evaluate medication administration, and parents will be asked to return the bottles with remaining antibiotic suspension at the final visit (which allows us to estimate the volume of the suspension actually administered).

We assume that once effectiveness and safety have been shown, implementation of intravenous-to-oral-switch-therapy will reduce the risks of hospital-related complications such as nosocomial infections and lead to an earlier return to a normal family setting with improvement in quality of life and better mother-child bonding. Furthermore we expect that the cost reduction will be substantial.

For peer review only

For peer review only - http://bmjopen.bmj.com/site/about/guidelines.xhtml

Author's contribution

GTS conceived the original idea for this trial. FK, RK, KM, MP, NH, KA and IR were involved in further conception and trial development. FK wrote the first draft. FK, GTS and RK rewrote the article. GTS, RK, KM, MP, NH, KA and IR critically revised the article for important intellectual content. All authors contributed to and approved the final version of the manuscript.

Funding statement

This work is supported by The Netherlands Organisation for Health Research and Development (ZonMW) grant number 848015005 and the Innovatiefonds Zorgverzekeraars.

Acknowledgements

We thank The Dutch parent organisation "Vereniging van Ouders van Couveusekinderen (VOC)" for their help in the conceptualization of the trial.

Competing interests

The authors declare that they have no competing interests.

Figure legends

1: Table 1: Inclusion and exclusion criteria RAIN study
2: **Figure 1: Flow chart of study procedures** [1]CRP: C-reactive protein; PCT: procalcitonin; [2]IV: intravenous

For peer review only - http://bmjopen.bmj.com/site/about/guidelines.xhtml

References

1. Cox LM, Yamanishi S, Sohn J, Alekseyenko AV, Leung JM, Cho I, et al. Altering the intestinal microbiota during a critical developmental window has lasting metabolic consequences. Cell. 2014;158(4):705-21.
2. Gray LE, O'Hely M, Ranganathan S, Sly PD, Vuillermin P. The Maternal Diet, Gut Bacteria, and Bacterial Metabolites during Pregnancy Influence Offspring Asthma. Front Immunol. 2017;8:365.
3. Johnson CC, Ownby DR. The infant gut bacterial microbiota and risk of pediatric asthma and allergic diseases. Transl Res. 2017;179:60-70.
4. Hyun DY, Hersh AL, Namtu K, Palazzi DL, Maples HD, Newland JG, et al. Antimicrobial stewardship in pediatrics: how every pediatrician can be a steward. JAMA Pediatr. 2013;167(9):859-66.
5. Fjalstad JW, Stensvold HJ, Bergseng H, Simonsen GS, Salvesen B, Ronnestad AE, et al. Early-onset Sepsis and Antibiotic Exposure in Term Infants: A Nationwide Population-based Study in Norway. Pediatr Infect Dis J. 2016;35(1):1-6.
6. Neonatal infection (early onset): antibiotics for prevention and treatment [Internet]. 2012 [cited 28-08-2018]. Available from: https://www.nice.org.uk/guidance/cg149.
7. African Neonatal Sepsis Trial g, Tshefu A, Lokangaka A, Ngaima S, Engmann C, Esamai F, et al. Simplified antibiotic regimens compared with injectable procaine benzylpenicillin plus gentamicin for treatment of neonates and young infants with clinical signs of possible serious bacterial infection when referral is not possible: a randomised, open-label, equivalence trial. Lancet. 2015;385(9979):1767-76.
8. Mir F, Nisar I, Tikmani SS, Baloch B, Shakoor S, Jehan F, et al. Simplified antibiotic regimens for treatment of clinical severe infection in the outpatient setting when referral is not possible for young infants in Pakistan (Simplified Antibiotic Therapy Trial [SATT]): a randomised, open-label, equivalence trial. Lancet Global Health. 2017;5(2):e177-e85.
9. Baqui AH, Saha SK, Ahmed AS, Shahidullah M, Quasem I, Roth DE, et al. Safety and efficacy of alternative antibiotic regimens compared with 7 day injectable procaine benzylpenicillin and gentamicin for outpatient treatment of neonates and young infants with clinical signs of severe infection when referral is not possible: a randomised, open-label, equivalence trial. Lancet Glob Health. 2015;3(5):e279-87.
10. A. Fuchs JB, S, Mathur, M. Sharland, J. N. Van Den Anker. Antibiotic Use for Sepsis in Neonates and Children: 2016 Evidence Update. WHO-Reviews. 2016.
11. Mir F. Thesis: High dose oral amoxicillin attains pharmacokinetic efficacy endpoints in young infants (0-59 days) with suspected sepsis - a population pharmacokinetic pilot study. EMORY Electronic Theses and Dissertations. 2013.
12. Autret E, Laugier J, Marimbu J, Vaillant MC, Furet Y, Breteau M. Comparison between IV and oral route amoxicillin administration in neonatal bacterial colonization. ARCH FR PEDIATR. 1988;45(9):679-82.
13. Gras-Le Guen C, Boscher C, Godon N, Caillon J, Denis C, Nguyen JM, et al. Therapeutic amoxicillin levels achieved with oral administration in term neonates. Eur J Clin Pharmacol. 2007;63(7):657-62.
14. Manzoni P, Esposito S, Gallo E, Gastaldo L, Farina D, Principi N. Switch therapy in full-term neonates with presumed or proven bacterial infection. J Chemother. 2009;21(1):68-73.
15. Chiesa C, Natale F, Pascone R, Osborn JF, Pacifico L, Bonci E, et al. C reactive protein and procalcitonin: reference intervals for preterm and term newborns during the early neonatal period. Clin Chim Acta. 2011;412(11-12):1053-9.
16. Chiesa C, Panero A, Rossi N, Stegagno M, De Giusti M, Osborn JF, et al. Reliability of procalcitonin concentrations for the diagnosis of sepsis in critically Ill neonates. Clin Infect Dis. 1998;26(3):664-72.
17. Green PA, Singh KV, Murray BE, Baker CJ. Recurrent group B streptococcal infections in infants: clinical and microbiologic aspects. J Pediatr. 1994;125(6 Pt 1):931-8.

For peer review only - http://bmjopen.bmj.com/site/about/guidelines.xhtml

18. Zangwill KM, Schuchat A, Wenger JD. Group B streptococcal disease in the United States, 1990: report from a multistate active surveillance system. MMWR CDC Surveill Summ. 1992;41(6):25-32.
19. Ekelund K, Konradsen HB. Invasive group B streptococcal disease in infants: a 19-year nationwide study. Serotype distribution, incidence and recurrent infection. Epidemiol Infect. 2004;132(6):1083-90.
20. Giannoni E, Agyeman PKA, Stocker M, Posfay-Barbe KM, Heininger U, Spycher BD, et al. Neonatal Sepsis of Early Onset, and Hospital-Acquired and Community-Acquired Late Onset: A Prospective Population-Based Cohort Study. J Pediatr. 2018;201:106-14 e4.
21. de Hoog M, Mouton JW, van den Anker JN. New dosing strategies for antibacterial agents in the neonate. Semin Fetal Neonatal Med. 2005;10(2):185-94.
22. EUCAST. European Committee on Antimicrobial Susceptibility Testing
Breakpoint tables for interpretation of MICs and zone diameters
Version 8.1. 2018.
23. Nederland Z. Guidelines for economic evaluations of health care. In: Institute NHC, editor.: National Health Care Institute; 2016.
24. Nederland Z. Manual for costing research. Methods and reference prices for economic evaluations in health care. Updated edition. National Health Care Institute. 2015.
25. Bouwmans C, Krol M, Severens H, Koopmanschap M, Brouwer W, Hakkaart-van Roijen L. The iMTA Productivity Cost Questionnaire: A Standardized Instrument for Measuring and Valuing Health-Related Productivity Losses. Value Health. 2015;18(6):753-8.
26. Stocker M, van Herk W, El Helou S, Dutta S, Fontana MS, Schuerman F, et al. Procalcitonin-guided decision making for duration of antibiotic therapy in neonates with suspected early-onset sepsis: a multicentre, randomised controlled trial (NeoPIns). Lancet. 2017;390(10097):871-81.

For peer review only - http://bmjopen.bmj.com/site/about/guidelines.xhtml

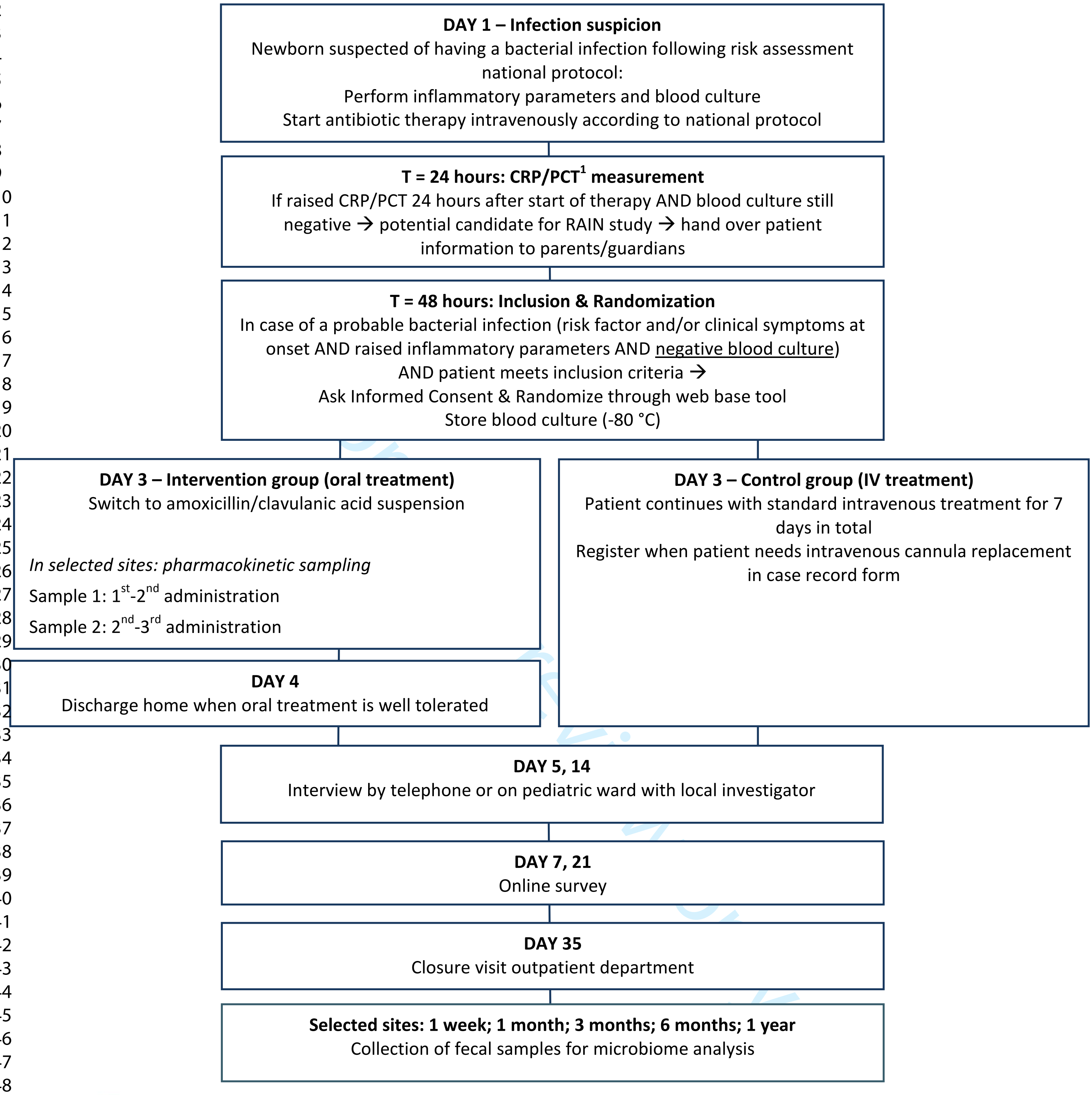

RAIN Reduction of intravenous Antibiotics In Neonates

Figure 1: Flow chart of study procedures [1]CRP: C-reactive protein; PCT: procalcitonin; [2]IV: intravenous

Supplementary material:

RAIN study: a protocol for a randomized controlled trial evaluating efficacy, safety and cost-effectiveness of intravenous-to-oral antibiotic switch therapy in neonates with a probable bacterial infection

1. SPIRIT checklist
2. Overview participating sites
3. Risk assessment tables for suspected early-onset bacterial infection from the Dutch national guideline: *NVK Richtlijn: Preventie en behandeling van early-onset neonatale infecties*
4. Questionnaires for assessment of cost-effectiveness (CEA) and Quality of Life (QoL).
5. Questionnaires for microbiome analysis

For peer review only

For peer review only - http://bmjopen.bmj.com/site/about/guidelines.xhtml

STANDARD PROTOCOL ITEMS: RECOMMENDATIONS FOR INTERVENTIONAL TRIALS

SPIRIT 2013 Checklist: Recommended items to address in a clinical trial protocol and related documents*

Section/item	Item No	Description	Page
**Administrative information**			
Title	1	Descriptive title identifying the study design, population, interventions, and, if applicable, trial acronym	1
Trial registration	2a	Trial identifier and registry name. If not yet registered, name of intended registry	2 & 15
	2b	All items from the World Health Organization Trial Registration Data Set	yes
Protocol version	3	Date and version identifier	yes
Funding	4	Sources and types of financial, material, and other support	18
Roles and responsibilities	5a	Names, affiliations, and roles of protocol contributors	1
	5b	Name and contact information for the trial sponsor	1
	5c	Role of study sponsor and funders, if any, in study design; collection, management, analysis, and interpretation of data; writing of the report; and the decision to submit the report for publication, including whether they will have ultimate authority over any of these activities	N/A
	5d	Composition, roles, and responsibilities of the coordinating centre, steering committee, endpoint adjudication committee, data management team, and other individuals or groups overseeing the trial, if applicable (see Item 21a for data monitoring committee)	13 & 14
**Introduction**			

For peer review only

For peer review only - http://bmjopen.bmj.com/site/about/guidelines.xhtml

Background and rationale	6a	Description of research question and justification for undertaking the trial, including summary of relevant studies (published and unpublished) examining benefits and harms for each intervention	4-6
	6b	Explanation for choice of comparators	4-6
Objectives	7	Specific objectives or hypotheses	6
Trial design	8	Description of trial design including type of trial (eg, parallel group, crossover, factorial, single group), allocation ratio, and framework (eg, superiority, equivalence, noninferiority, exploratory)	7

Methods: Participants, interventions, and outcomes

Study setting	9	Description of study settings (eg, community clinic, academic hospital) and list of countries where data will be collected. Reference to where list of study sites can be obtained	7
Eligibility criteria	10	Inclusion and exclusion criteria for participants. If applicable, eligibility criteria for study centres and individuals who will perform the interventions (eg, surgeons, psychotherapists)	7
Interventions	11a	Interventions for each group with sufficient detail to allow replication, including how and when they will be administered	8
	11b	Criteria for discontinuing or modifying allocated interventions for a given trial participant (eg, drug dose change in response to harms, participant request, or improving/worsening disease)	8
	11c	Strategies to improve adherence to intervention protocols, and any procedures for monitoring adherence (eg, drug tablet return, laboratory tests)	10
	11d	Relevant concomitant care and interventions that are permitted or prohibited during the trial	N/A

For peer review only - http://bmjopen.bmj.com/site/about/guidelines.xhtml

Outcomes	12	Primary, secondary, and other outcomes, including the specific measurement variable (eg, systolic blood pressure), analysis metric (eg, change from baseline, final value, time to event), method of aggregation (eg, median, proportion), and time point for each outcome. Explanation of the clinical relevance of chosen efficacy and harm outcomes is strongly recommended	11
Participant timeline	13	Time schedule of enrolment, interventions (including any run-ins and washouts), assessments, and visits for participants. A schematic diagram is highly recommended (see Figure)	Figure is added
Sample size	14	Estimated number of participants needed to achieve study objectives and how it was determined, including clinical and statistical assumptions supporting any sample size calculations	10
Recruitment	15	Strategies for achieving adequate participant enrolment to reach target sample size	N/A
**Methods: Assignment of interventions (for controlled trials)**			
Allocation:			
Sequence generation	16a	Method of generating the allocation sequence (eg, computer-generated random numbers), and list of any factors for stratification. To reduce predictability of a random sequence, details of any planned restriction (eg, blocking) should be provided in a separate document that is unavailable to those who enrol participants or assign interventions	8
Allocation concealment mechanism	16b	Mechanism of implementing the allocation sequence (eg, central telephone; sequentially numbered, opaque, sealed envelopes), describing any steps to conceal the sequence until interventions are assigned	8

Implementation	16c	Who will generate the allocation sequence, who will enrol participants, and who will assign participants to interventions	8
Blinding (masking)	17a	Who will be blinded after assignment to interventions (eg, trial participants, care providers, outcome assessors, data analysts), and how	N/A, page 8
	17b	If blinded, circumstances under which unblinding is permissible, and procedure for revealing a participant's allocated intervention during the trial	N/A
**Methods: Data collection, management, and analysis**			
Data collection methods	18a	Plans for assessment and collection of outcome, baseline, and other trial data, including any related processes to promote data quality (eg, duplicate measurements, training of assessors) and a description of study instruments (eg, questionnaires, laboratory tests) along with their reliability and validity, if known. Reference to where data collection forms can be found, if not in the protocol	11
	18b	Plans to promote participant retention and complete follow-up, including list of any outcome data to be collected for participants who discontinue or deviate from intervention protocols	N/A
Data management	19	Plans for data entry, coding, security, and storage, including any related processes to promote data quality (eg, double data entry; range checks for data values). Reference to where details of data management procedures can be found, if not in the protocol	11
Statistical methods	20a	Statistical methods for analysing primary and secondary outcomes. Reference to where other details of the statistical analysis plan can be found, if not in the protocol	12
	20b	Methods for any additional analyses (eg, subgroup and adjusted analyses)	12
	20c	Definition of analysis population relating to protocol non-adherence (eg, as randomised analysis), and any statistical methods to handle missing data (eg, multiple imputation)	11

Methods: Monitoring

Data monito ring	21a	Composition of data monitoring committee (DMC); summary of its role and reporting structure; statement of whether it is independent from the sponsor and competing interests; and reference to where further details about its charter can be found, if not in the protocol. Alternatively, an explanation of why a DMC is not needed	12 & 16
	21b	Description of any interim analyses and stopping guidelines, including who will have access to these interim results and make the final decision to terminate the trial	11-12
Harms	22	Plans for collecting, assessing, reporting, and managing solicited and spontaneously reported adverse events and other unintended effects of trial interventions or trial conduct	16
Auditin g	23	Frequency and procedures for auditing trial conduct, if any, and whether the process will be independent from investigators and the sponsor	N/A

Ethics and dissemination

Resear ch ethics approv al	24	Plans for seeking research ethics committee/institutional review board (REC/IRB) approval	17
Protoc ol amend ments	25	Plans for communicating important protocol modifications (eg, changes to eligibility criteria, outcomes, analyses) to relevant parties (eg, investigators, REC/IRBs, trial participants, trial registries, journals, regulators)	N/A
Conse nt or assent	26a	Who will obtain informed consent or assent from potential trial participants or authorised surrogates, and how (see Item 32)	7-8
	26b	Additional consent provisions for collection and use of participant data and biological specimens in ancillary studies, if applicable	Mentioned in patient informatio n form

For peer review only - http://bmjopen.bmj.com/site/about/guidelines.xhtml

Confidentiality	27	How personal information about potential and enrolled participants will be collected, shared, and maintained in order to protect confidentiality before, during, and after the trial	Mentioned in patient information form, page 14
Declaration of interests	28	Financial and other competing interests for principal investigators for the overall trial and each study site	20
Access to data	29	Statement of who will have access to the final trial dataset, and disclosure of contractual agreements that limit such access for investigators	A trial agreement has been made. Not mentioned in the manuscript.
Ancillary and post-trial care	30	Provisions, if any, for ancillary and post-trial care, and for compensation to those who suffer harm from trial participation	Mentioned in patient information form
Dissemination policy	31a	Plans for investigators and sponsor to communicate trial results to participants, healthcare professionals, the public, and other relevant groups (eg, via publication, reporting in results databases, or other data sharing arrangements), including any publication restrictions	16-17
	31b	Authorship eligibility guidelines and any intended use of professional writers	N/A
	31c	Plans, if any, for granting public access to the full protocol, participant-level dataset, and statistical code	N/A
**Appendices**			

For peer review only - http://bmjopen.bmj.com/site/about/guidelines.xhtml

Informed consent materials	32	Model consent form and other related documentation given to participants and authorised surrogates	Not attached, in dutch.
Biological specimens	33	Plans for collection, laboratory evaluation, and storage of biological specimens for genetic or molecular analysis in the current trial and for future use in ancillary studies, if applicable	12-15

For peer review only

For peer review only - http://bmjopen.bmj.com/site/about/guidelines.xhtml

2. Table 1: participating sites RAIN study

Amphia Hospital	Breda, The Netherlands
Erasmus MC-Sophia Children's Hospital	Rotterdam, The Netherlands
Groene Hart Hospital	Gouda, The Netherlands
Haaglanden Medical Center	Den Haag, The Netherlands
Haga/Juliana Children's Hospital	Den Haag, The Netherlands
Ikazia Hospital	Rotterdam, The Netherlands
Isala Klinieken	Zwolle, The Netherlands
Maasstad Hospital	Rotterdam, The Netherlands
Maxima Medical Center	Veldhoven, The Netherlands
Meander Medical Center	Amersfoort, The Netherlands
Medisch Spectrum	Twente, The Netherlands
Reinier de Graaf Hospital	Delft, The Netherlands
Rijnstate Hospital	Arnhem, The Netherlands
Sint Antonius Hospital *(2 locations)*	Nieuwegein & Utrecht, The Netherlands
Franciscus Gasthuis & Vlietland *(2 locations)*	Rotterdam & Schiedam, The Netherlands
IJsselland Hospital	Capelle aan den IJssel, The Netherlands

For peer review only

For peer review only - http://bmjopen.bmj.com/site/about/guidelines.xhtml

3. Risk assessment tables for suspected early-onset bacterial infection from the Dutch national guideline: *NVK Richtlijn: Preventie en behandeling van early-onset neonatale infecties*

Table 1: Risk factors for early-onset neonatal infection, including 'red flags

Risk factor	Red flag
**Parenteral antibiotic treatment given to the woman for confirmed or suspected invasive bacterial infection (such as septicaemia) at any time during labour, or in the 24-hour periods before and after the birth [This does not refer to intrapartum antibiotic prophylaxis]**	yes
**Suspected or confirmed infection in another baby in the case of a multiple pregnancy**	yes
Invasive group B streptococcal infection in a previous baby	
Maternal group B streptococcal colonisation, bacteriuria or infection in the current pregnancy	
Prelabour rupture of membranes >24 hours in term baby	
Preterm birth following spontaneous labour (before 37 weeks' gestation)	
Suspected or confirmed rupture of membranes for more than 18 hours in a preterm birth	
Intrapartum fever higher than 38°C, or confirmed or suspected chorioamnionitis	

Table 2 Clinical indicators of possible early-onset neonatal infection (observations and events in the baby), including 'red flags' Dutch national protocol. Adopted from the NICE guidelines 2012.

Clinical indicator	Red flag
**Respiratory distress starting more than 4 hours after birth**	yes
**Neonatal seizures**	yes
**Need for mechanical ventilation in a term baby**	yes
**Signs of shock**	yes
Altered behaviour or responsiveness (including floppiness)	
Feeding difficulties (including vomiting, excessive gastric aspirates and abdominal distension)	
Apnoea or bradycardia	
Signs of respiratory distress	
Hypoxia (for example, central cyanosis or reduced oxygen saturation level	
Signs of neonatal encephalopathy	
Need for cardio–pulmonary resuscitation	
Need for mechanical ventilation in a preterm baby	
Persistent fetal circulation (persistent pulmonary hypertension)	
Temperature abnormality (lower than 36°C or higher than 38°C) unexplained by environmental factors	
Signs of local infection (eyes, skin)	

<u>*References:*</u>

1. Neonatal infection (early onset): antibiotics for prevention and treatment [Internet]. 2012 [cited 28-08-2018]. Available from: https://www.nice.org.uk/guidance/cg149.

2. NVK Richtlijn: Preventie en behandeling van early-onset neonatale infecties, 2017. [cited 23-01-2019] Available from: https://www.nvk.nl/Kwaliteit/Richtlijnen-overzicht/Details/articleType/ArticleView/articleId/694/Preventie-en-behandeling-van-early-onset-neonatale-infecties#tab0

For peer review only - http://bmjopen.bmj.com/site/about/guidelines.xhtml

Survey 'ENGLISH: Survey day 7'

ENGLISH: Survey day 7 - Introduction

Dear parents/guardians, We have asked you permission to send two surveys as follow up on the RAIN study. This is the first survey, which has to be filled in around day 7 after initiation of therapy. Thank you for your help! RAIN team

ENGLISH: Survey day 7 - Survey day 7

Number	Question	Answers
1.1	Age mother	[ ] years
1.2	Age father	[ ] years
1.3	What is your highest degree in education?	○ I never finished school or training programme<br>○ Primary/Elementary school<br>○ Junior vocational eductaion<br>○ Lower genearal secondary education<br>○ Intermediate vocational education<br>○ Higher general secundary education<br>○ School for higher vocational education<br>○ University<br>○ Other degree
1.3.1	***If 'What is your highest degree in education?' is equal to 'Other degree' answer this question:***<br>If other, specify degree	[ ]
1.4	What is your highest degree in education?	○ I never finished school or training programme<br>○ Primary/Elementary school<br>○ Junior vocational eductaion<br>○ Lower genearal secondary education<br>○ Intermediate vocational education<br>○ Higher general secundary education<br>○ School for higher vocational education<br>○ University<br>○ Other degree
1.3.2	***If 'What is your highest degree in education?' is equal to 'Other degree' answer this question:***<br>If other, specify degree	[ ]

1.5	How many children (<18 years) do you have?	[ ] Children
1.6	Do they all live at home with you?	○ Yes<br>○ No
1.7	Do you, mother, have a paid job?	○ No<br>○ Yes, fulltime (>36 hours/week)<br>○ Yes, parttime
1.7.1	***If 'Do you, mother, have a paid job?' is equal to 'Yes, parttime' answer this question:***<br>If parttime, how many hours per week?	[ ] hours
1.7.2	***If 'Do you, mother, have a paid job?' is not equal to 'No' answer this question:***<br>Mother: What is (on average) your monthly salary (gross amount)?	○ Less than €999/month<br>○ Between €1.000 and 1.499/month<br>○ Between €1.500 and 1.999/month<br>○ Between €2.000 and 2.499/month<br>○ Between €2.500 and 2.999/month<br>○ Between €3.000 and 3.499/month<br>○ Between €3.500 and 3.999/month<br>○ Between €4.000 and 4.999/month<br>○ Between €5.000 and 5.999/month<br>○ Between €6.000 and 6.999/month<br>○ Between €7.000 and 7.999/month<br>○ Between €8.000 and 8.999/month<br>○ Between €9.000 and 9.999/month<br>○ More than €10.000/month<br>○ I don't know, I do not want to tell this
1.8	Do you, mother, have an unpaid job (except for caregiving)?	○ Yes<br>○ No
1.8.1	***If 'Do you, mother, have an unpaid job (except for caregiving)?' is equal to 'Yes' answer this question:***<br>if yes, for how many hours per month?	[ ]
1.9	Do you, father, have a paid job?	○ No<br>○ Yes, fulltime (>36 hours/week)<br>○ Yes, parttime
1.9.1	***If 'Do you, father, have a paid job?' is equal to 'Yes, parttime' answer this question:***<br>If parttime, how many hours per week?	[ ] hours

For peer review only - http://bmjopen.bmj.com/site/about/guidelines.xhtml

1.9.2	***If 'Do you, father, have a paid job?' is not equal to 'No' answer this question:*** Father: What is (on average) your monthly salary (gross amount)?	Less than €999/month<br>Between €1.000 and 1.499/month<br>Between €1.500 and 1.999/month<br>Between €2.000 and 2.499/month<br>Between €2.500 and 2.999/month<br>Between €3.000 and 3.499/month<br>Between €3.500 and 3.999/month<br>Between €4.000 and 4.999/month<br>Between €5.000 and 5.999/month<br>Between €6.000 and 6.999/month<br>Between €7.000 and 7.999/month<br>Between €8.000 and 8.999/month<br>Between €9.000 and 9.999/month<br>More than €10.000/month<br>I don't know, I do not want to tell this
1.10	Do you, father, have an unpaid job (except for caregiving)?	Yes<br>No
1.10.1	***If 'Do you, father, have an unpaid job (except for caregiving)?' is equal to 'Yes' answer this question:*** Father: if yes, for how many hours per month?	
1.11	Did you hire extra baby-sitting because of the hospital admission of your child?	Yes<br>No
1.12	Did you partner, in the past week, work less than expected in order for him tot take care of you and/or other children because of the admission?	Yes<br>No
1.12.1	***If 'Did you partner, in the past week, work less than expected in order for him tot take care of you and/or other children because of the admission?' is equal to 'Yes' answer this question:*** If yes, how many hours less than expected?	

1.13	For how many days was your baby admitted to the hospital?	○ 2.5 days<br>○ 3 days<br>○ 3.5 days<br>○ 4 days<br>○ 4.5 days<br>○ 5 days<br>○ 5.5 days<br>○ 6 days<br>○ 6.5 days<br>○ 7 days<br>○ 7.5 days<br>○ 8 days<br>○ > 8 days
1.14	How many times did your baby receive a new IV canula?	○ Not, because he/she was treated with antibiotic suspension<br>○ 1 x<br>○ 2 x<br>○ 3 x<br>○ 4 x<br>○ More often<br>○ I do not remember<br>○ My child only kept the first inserted canula for the whole treatment
1.15	How high were your travel expenses each day for travelling from your house to the hospital?	[ ] euro
1.16	Did you visit another doctor this week for your baby?	○ Yes<br>○ No
1.16.1	***If 'Did you visit another doctor this week for your baby?' is equal to 'Yes' answer this question:***<br>If yes, what kind of doctor?	[ ]
1.17	Did your baby experience fever this week?	○ Yes<br>○ No
1.17.1	***If 'Did your baby experience fever this week?' is equal to 'Yes' answer this question:***<br>If yes, how high was the fever?	[ ] degrees Celcius

1.18	Did your child experience any side effects of the antibiotic therapy?	Vomiting<br>Change is defecation pattern<br>Rash<br>Other<br>None
1.19	What kind of feeding do you give to your baby?	Breastmilk<br>Formula milk<br>Combination of breast- and formulamilk
1.20	How does your child sleep?	Goes to sleep easily and wakes up satisfied<br>Going to sleep is sometimes difficult, but does eventually sleep well<br>Going to sleep is difficult, cries often, hard to comfort
1.21	Did your baby receive any other medication (besides vitamins)?	Yes<br>No
1.21.1	***If 'Did your baby receive any other medication (besides vitamins)?' is equal to 'Yes' answer this question:***<br>If yes, which medication?	

ENGLISH: Survey day 7 - Outro

Thank you for helping us by filling in this survey. You will receive the second survey in two weeks. Kind regards, RAIN team

Survey 'ENGLISH survey day 21'

ENGLISH survey day 21 - Introduction

Dear parents/guardians, We have asked you permission to send two surveys as follow up on the RAIN study. This is the second survey, which has to be filled in around 3 weeks after initiation of therapy. Thank you for your help! RAIN team

ENGLISH survey day 21 - ENGLISH survey day 21

Number	Question	Answers
1.1	Did you visit another doctor this week for your baby?	○ Yes<br>○ No
1.1.1	***If 'Did you visit another doctor this week for your baby?' is equal to 'Yes' answer this question:***<br>If yes, what kind of doctor?	[ ]
1.2	Did your baby experience fever this week?	○ Yes<br>○ No
1.2.1	***If 'Did your baby experience fever this week?' is equal to 'Yes' answer this question:***<br>If yes, how high was the fever?	[ ] degrees Celcius
1.3	What kind of feeding do you give to your baby?	○ Breastmilk<br>○ Formula milk<br>○ Combination of breast- and formulamilk
1.4	How does your child sleep?	○ Goes to sleep easily and wakes up satisfied<br>○ Going to sleep is sometimes difficult, but does eventually sleep well<br>○ Going to sleep is difficult, cries often, hard to comfort
1.5	Did your baby receive any other medication (besides vitamins)?	○ Yes<br>○ No
1.5.1	***If 'Did your baby receive any other medication (besides vitamins)?' is equal to 'Yes' answer this question:***<br>If yes, which medication?	[ ]

ENGLISH survey day 21 - Outro

Thank you for helping us by filling in this survey. This was the last survey of the RAIN study. Kind regards, RAIN team

For peer review only - http://bmjopen.bmj.com/site/about/guidelines.xhtml

Survey 'ENGLISH: microbiome T = 6 months'

ENGLISH: microbiome T = 6 months - Introduction

Dear parents/guardians, You have agreed to collect fecal samples in the first year of life of your child. It is time to collect a sample for the 4th time. 1. Please collect some stool from the diaper of your baby in the tube with the label: RAIN FECES 4 T = 6 months and put it directly back into the freezer. 2. Please answer the following questions.

ENGLISH: microbiome T = 6 months - questionnaire microbiome T = 6 months

Number	Question	Answers
1.1	What kind of feeding do you give to your child?	○ Breastmilk<br>○ Formula milk<br>○ Combination of breast- and formulamilk
1.2	Did you child start with solid food yet?	○ Yes<br>○ No
1.3	Are there older children living with you and your child in the same house?	○ no<br>○ yes, 1<br>○ yes, 2<br>○ yes, 3<br>○ yes, more than 3
1.3.1	***If 'Are there older children living with you and your child in the same house?' is not equal to 'no' answer this question:***<br>If yes, what age group are they in?	☐ < 4 years old<br>☐ 4-12 years old<br>☐ >12 years old
1.3.1.1	***If 'If yes, what age group are they in?' is equal to '< 4 years old' answer this question:***<br>Are your children, younger than 4 years old, going to daycare?	○ No<br>○ Yes, 1 day /week<br>○ Yes, 2 days /week<br>○ Yes, more than 2 days/week

1.4	Did your child experience any episodes of illness in the last 3 months?	☐ No<br>☐ Yes, nasal congestion/stuffy nose<br>☐ Yes, coughing<br>☐ Yes, fever > 38.0 degrees celcius<br>☐ Yes, diarrhea<br>☐ Yes, skin rash for more than 1 day
1.4.1	***If 'Did your child experience any episodes of illness in the last 3 months?' is not equal to 'No' answer this question:***<br>If yes, did you visit a doctor?	○ Yes<br>○ No
1.4.1.1	***If 'If yes, did you visit a doctor?' is equal to 'Yes' answer this question:***<br>If yes, please specify who (example: general practitioner, pediatrician)	
1.5	Has your child been readmitted to the hospital in the last 3 months?	○ Yes<br>○ No
1.5.1	***If 'Has your child been readmitted to the hospital in the last 3 months?' is equal to 'Yes' answer this question:***<br>If yes, for what reason?	
1.6	Did your child receive another course of antibiotics in the past 3 months?	○ Yes<br>○ No
1.6.1	***If 'Did your child receive another course of antibiotics in the past 3 months?' is equal to 'Yes' answer this question:***<br>If you remember the name of the treatment, please write it down	

ENGLISH: microbiome T = 6 months - Outro

In 6 months you will receive the last questionnaire and collect the last stool sample. Thank you for your help! RAIN team

For peer review only - http://bmjopen.bmj.com/site/about/guidelines.xhtml
